# Octreotide LAR and Prednisone as Neoadjuvant Treatment in Patients with Primary or Locally Recurrent Unresectable Thymic Tumors: A Phase II Study

**DOI:** 10.1371/journal.pone.0168215

**Published:** 2016-12-16

**Authors:** Lukas Kirzinger, Sandra Boy, Jörg Marienhagen, Gerhard Schuierer, Reiner Neu, Michael Ried, Hans-Stefan Hofmann, Karsten Wiebe, Philipp Ströbel, Christoph May, Julia Kleylein-Sohn, Claudia Baierlein, Ulrich Bogdahn, Alexander Marx, Berthold Schalke

**Affiliations:** 1 Department of Neurology, University of Regensburg, Regensburg, Germany; 2 Department of Nuclear Medicine, University of Regensburg, Regensburg, Germany; 3 Institute of Neuroradiology, BKR, Regensburg, Germany; 4 Department of Thoracic Surgery, University of Regensburg, Regensburg, Germany; 5 Department of Cardiac and Thoracic Surgery, University of Muenster, Muenster, Germany; 6 Institute of Pathology, University of Goettingen, Goettingen, Germany; 7 Novartis Pharma GmbH, Nuremberg, Germany; 8 Institute of Pathology, University Medical Centre Mannheim, University of Heidelberg, Mannheim, Germany; Gustave Roussy, FRANCE

## Abstract

Therapeutic options to cure advanced, recurrent, and unresectable thymomas are limited. The most important factor for long-term survival of thymoma patients is complete resection (R0) of the tumor. We therefore evaluated the response to and the induction of resectability of primarily or locally recurrent unresectable thymomas and thymic carcinomas by octreotide Long-Acting Release (LAR) plus prednisone therapy in patients with positive octreotide scans. In this open label, single-arm phase II study, 17 patients with thymomas considered unresectable or locally recurrent thymoma (n = 15) and thymic carcinoma (n = 2) at Masaoka stage III were enrolled. Octreotide LAR (30 mg once every 2 weeks) was administered in combination with prednisone (0.6 mg/kg per day) for a maximum of 24 weeks (study design according to Fleming´s one sample multiple testing procedure for phase II clinical trials). Tumor size was evaluated by volumetric CT measurements, and a decrease in tumor volume of at least 20% at week 12 compared to baseline was considered as a response. We found that octreotide LAR plus prednisone elicited response in 15 of 17 patients (88%). Median reduction of tumor volume after 12 weeks of treatment was 51% (range 20%–86%). Subsequently, complete surgical resection was achieved in five (29%) and four patients (23%) after 12 and 24 weeks, respectively. Octreotide LAR plus prednisone treatment was discontinued in two patients before week 12 due to unsatisfactory therapeutic effects or adverse events. The most frequent adverse events were gastrointestinal (71%), infectious (65%), and hematological (41%) complications. In conclusion, octreotide LAR plus prednisone is efficacious in patients with primary or recurrent unresectable thymoma with respect to tumor regression. Octreotide LAR plus prednisone was well tolerated and adverse events were in line with the known safety profile of both agents.

## Introduction

Thymomas originate from the epithelial cells of the thymus and almost invariably retain thymic characteristics such as the presence of intratumorous thymocytes, which is in contrast to other cancers including thymic carcinomas [1]. This property is likely the basis for the high frequency of autoimmune diseases, in particular Myasthenia gravis [2], as well as immunodeficiency states associated with thymomas [3]. Although thymomas are rare (incidence: 0.15 cases per 100.000 persons per year), they are the most common primary malignancy of the anterior mediastinum in adults [4, 5]. Based on epithelial cell morphology and thymocyte content, the WHO histologically classifies epithelial thymic tumors into A, AB, B1, B2, and B3 thymomas as well as thymic carcinoma [6]. Many studies have demonstrated that the WHO classification is an independent prognostic marker [7, 8]. However, Masaoka tumor staging (stage I-IV) according to local invasiveness and expansion [9] as well as resection status [10–13] appear to be more relevant.

Surgery is the mainstay of thymoma therapy. Since incomplete resection of thymomas and thymic carcinomas (R1 and R2) is an adverse prognostic factor, achievement of complete resection (R0) is of upmost importance. Incomplete resection significantly influences survival, with a 5-year survival rate of 55% and 48% in R1- and R2-resected tumors, respectively, in contrast to 70% in R0 tumors [14, 15]. Surgery alone appears to be sufficient in approximately 50% of low risk thymoma patients [10, 16].

Patients with advanced disease (stage III or IV) and unresectable or recurrent tumors usually receive chemotherapy [17]. The CAP-regimen (cisplatin, doxorubicin, cyclophosphamide [18]) achieves response rates of 50% and has long been considered as standard first-line adjuvant chemotherapy [17]. For thymic carcinoma, multimodal treatment has become the preferred approach [19]. Because patients are usually not cured by chemotherapy, neoadjuvant multimodality approaches appear promising and worth testing for unresectable thymoma. So far, however, only small series and one prospective study have been reported [20–22]. Therefore, management is severely hampered by the lack of established alternatives once the standard first-line chemotherapy has failed, and there is only a limited number of reports on promising targeted therapies [17, 23].

Octreotide has pharmacologic effects similar to those of the natural hormone somatostatin and is an even more potent inhibitor of growth hormone, glucagon, and insulin than somatostatin [24]. It has been used to treat symptoms associated with both metastatic neuroendocrine tumors and vasoactive intestinal peptide (VIP) secreting adenomas. In patients with acromegaly, octreotide was shown to substantially reduce or even normalize growth hormone and/or insulin-like growth factor 1 levels [25]. Additionally, an anti-proliferative effect of somatostatin has been demonstrated in various tumors through the inhibition of angiogenesis and growth factors such as the insulin-like growth factor 1 [24–28].

Among the five subtypes of the somatostatin receptor (SSR), code-named sst1–5, sst2 is commonly detected in various tumor tissues [29]. SSRs are also expressed in thymoma [30, 31], whereas expression of somatostatin is missing. Imaging of SSR expression by using the radiolabeled somatostatin analog octreotide is a diagnostic tool to assess malignant thymic masses and metastasis [32]. In addition, successful somatostatin analogue therapy has been observed in cases of advanced, heavily pretreated, and unresectable thymoma, in which treatment with octreotide and prednisone resulted in complete remission or tumor size reduction [33, 34]. These observations were confirmed by a small phase II study on 16 patients with advanced refractory thymic tumors, demonstrating an overall response rate of 37% and one (6%) complete response [35]. Another phase II study demonstrated an overall response rate of 30% in 38 patients with advanced metastatic disease who had undergone prior surgery, radiation therapy, or chemotherapy in most cases [36]. Response to third-line palliative octreotide therapy was also demonstrated by a case report on a patient with recurrent unresectable thymoma [25].

Based on these positive observations, we initiated a phase II trial (NCT00332969) in patients with primarily unresectable or advanced locally recurrent thymoma or thymic carcinoma to evaluate the objective response rate, surgical resectability following response, and the occurrence of Myasthenia gravis after octreotide plus prednisone treatment.

## Patients and Methods

The study was approved by the Ethics Committee of the University of Regensburg. (Nr. 04/154) on 07/02/2005. Patients had to be older than 18 years and had to sign a written informed consent. Unfortunately, the registration of the study in the ClinicalTrials.gov registry was delayed due to organizational reasons. The authors confirm that all ongoing and related trials for this drug/intervention are registered.

### Patients

Patients with histologically confirmed thymic epithelial tumors of all WHO subtypes were eligible if thymomas were locally advanced or recurrent and/or thymic tumors were inoperable. Inoperability of thymic tumors was defined as the inability to achieve a complete en-bloc resection with definite clear margins (R0) due to at least adherence of the tumor to the neighboring organs and/or suspected tumor infiltration into neighboring organs and/or local recurrence of the thymic tumor. This judgement had to be made by a surgeon and a radiologist based on the computed tomography (CT)-scans and optional further imaging (magnetic resonance imaging). Signs of inoperability were tumor infiltration, more than 50% abutment of the vessel circumference, broad adherence of the tumor to the neighboring organs, or suspected tumor infiltration into neighboring organs including the aorta, vena cava, cardiac structures, trachea, and the thoracic wall. Infiltrations of the lung, the pericardium, or the brachiocephalic vein were considered resectable. All patients had to have a positive octreoscan, an ECOG (Eastern Cooperative Oncology Group [37]) performance status of 0, 1 or 2, and adequate hepatic function with alanine aminotransferase, aspartate aminotransferase, alkaline phosphatase <2x or total bilirubin <1.5x upper limit of normal. Patients were required to demonstrate tolerance to a test-dose of 0.1 mg subcutaneous octreotide received at visit 1 to ensure tolerability of subsequent treatment. Patients were not eligible if they had symptomatic cholelithiasis, grade III or IV cardiac disease as defined by the New York Heart Association Criteria, or severe or uncontrolled medical disease (such as uncontrolled diabetes, chronic renal disease, or infection). Patients had to be older than 18 years and had to sign a written informed consent. The clinical study was designed, implemented, and reported in accordance with the ICH Harmonized Tripartite Guidelines for Good Clinical Practice, with applicable local regulations (including European Directive 2001/83/EC and US Code of Federal Regulations Part 21), and with the ethical principles laid down in the Declaration of Helsinki. Participant recruitment was started in September 2005, the last patient completed in October 2010.

### Treatment

Patients received intramuscular injections of 30 mg octreotide Long-Acting Release (LAR) every two weeks and oral 0.6 mg/kg prednisone per day, for a maximum of 24 weeks. After 12 weeks, an interim evaluation of resectability of the tumor was performed on the basis of CT-scans at week 6 and 12. Patients who reached operability were treated until the date of surgery, which was performed 4 weeks after the end of study visit at the latest. Patients who had persistently unresectable tumors continued to receive octreotide LAR plus prednisone for additional 12 weeks. In case of adverse events (AEs), dose reduction of octreotide LAR to 20 mg was allowed.

### Evaluation

Before treatment, data on histology, medical history, physical examinations, blood values (hemoglobin, hematocrit, platelet and leucocyte counts, differential leucocyte count, metabolic profile) and serum titin and serum acetylcholine receptor (AChR) antibody levels were obtained, and thorax CT scans, electrocardiogram (ECG) and gallbladder ultrasound evaluations, as well as Myasthenia gravis scoring according to Besinger were performed [38]. At week 6, 12, 18, and 24, physical examination, CT scans, assessment of the metabolic profile, serum titin and serum ACHR antibodies, as well as gallbladder ultrasound examination and Myasthenia gravis scoring were performed prior to octreotide LAR administration.

Tumor volumes were determined by contrast CT imaging using the „Volume“-software package of the CT-scanner (Siemens-Medical, Forchheim Germany). The target outline was manually delineated on sequential CT slices, which was supported by a shape-propagation algorithm. Then, the volume of the tumor was calculated using cut-off values of 0 Hounsfield units and 150 Hounsfield units to exclude fat-tissue and large vessels from the data. This method provides reasonable correct tumor volumes for comparison of serial CT-studies.

### Design and statistical analysis

The primary objective of the study was to evaluate the efficacy of octreotide LAR in the treatment of inoperable primary and/or locally recurrent thymic tumors with respect to the shrinkage of tumor size. A response was defined as the decrease in tumor volume of at least 20% at week 12 as compared to baseline. Secondary objectives included the evaluation of tumor shrinkage to reach operability, resection status after 12 and 24 weeks of treatment, as well as safety and tolerability of octreotide LAR. Safety and tolerability analyses were performed on the safety population comprising all patients who had received at least one dose of study medication. The per protocol population comprised all patients who had received at least one dose of study medication and for whom at least one post baseline measurement of tumor volume was available. For this population, confirmatory analysis of the primary efficacy variable and analysis of secondary parameters were performed.

This study was designed according to Fleming’s one-sample multiple testing procedure for phase II clinical trials [39]. A total of 25 patients were planned to be recruited at two stages. At the first stage, 15 patients were planned to be recruited. Based on the number of responders, it was planned to recruit additional 10 patients for a second stage, if the trial was not to be stopped earlier due to futility or success. A response rate of less than or equal to 20% was considered not to warrant further investigation of the drug, whereas a response rate of at least 40% was considered to warrant further investigation.

## Results

### Patient Recruitment

**Fig 1:**
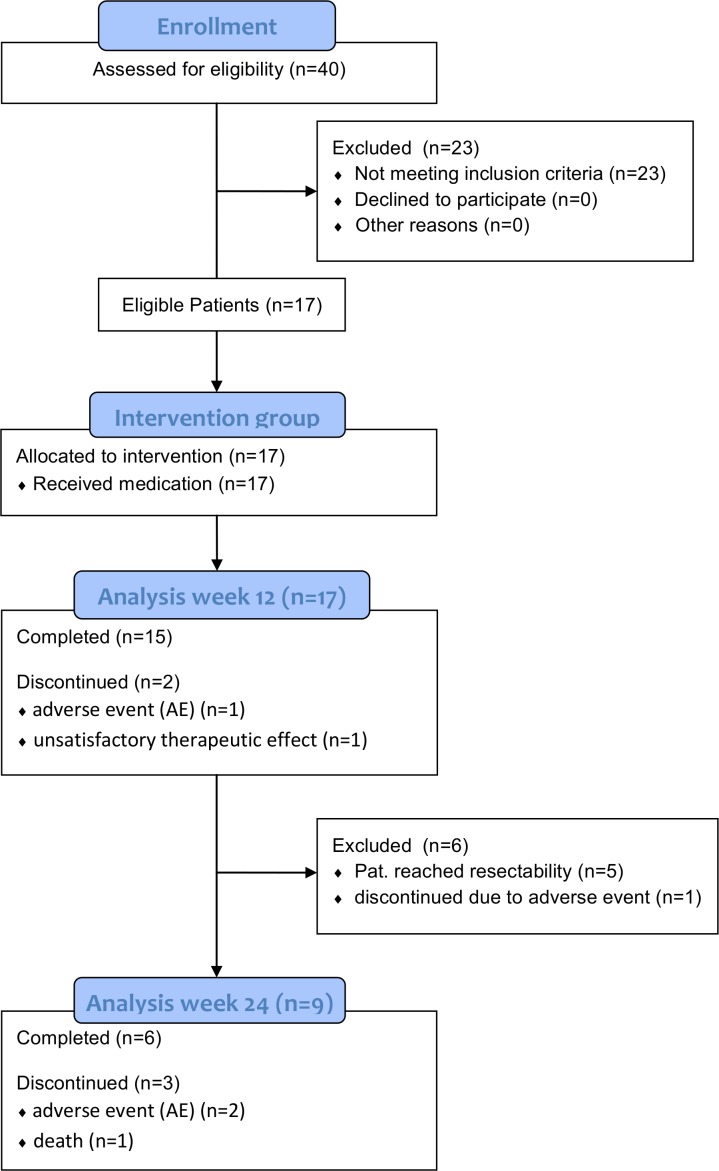
Study flow chart. A total of 17 patients were enrolled, of which 15 and 6 patients completed the first and the second 12-week-phase of treatment, respectively. Nine patients reached surgical radical resectability after 12 weeks (n = 5) and 24 weeks (n = 4).

Between September 2005 and October 2010, 17 patients with primary or locally recurrent unresectable thymic tumors were included. According to the study protocol, an interim analysis was performed after recruitment of 15 patients. In accordance with Fleming`s one-sample testing procedure for phase II clinical trials [39], the recruitment could have been stopped after recruitment of sufficient subjects, but two additional patients who had no other treatment options were included at the time of the interim analysis. We decided to include these patients into the final analysis (Fig 1).

### Patient Characteristics

Patient characteristics are described in Table 1. Patients were 65.2 ± 14.0 years old (mean ± SD), and the majority of patients was female (76%). All patients were Caucasians. The ECOG performance score was 0 and 1 for the majority of patients (eight patients each; ECOG score = 2 in one patients). All patients had a presumed stage III thymic tumor according to the Masaoka staging system, and patients were classified into WHO thymoma subtypes B2 (seven patients), B3 (five patients), and AB (three patients). Two patients had thymic carcinoma, which were classified as squamous cell carcinoma. The median time between first diagnosis and treatment was 0.3 years. For 88% of patients, it was less than one year. At study start, six patients were positive for antibodies against the AChR but showed no clinically relevant symptoms of myasthenia gravis (Table 2).

**Table 1:** Patient characteristics.

Variable		Total (N = 17)
**Age (years)**		
	mean (SD)	65.2 (14.0)
	range	38–84
**Sex, n (%)**		
	male	4 (24)
	female	13 (76)
**Race, n (%)**		
	caucasian	17 (100)
	other	0 (0)
**ECOG performance score, n (%)**		
	0	8 (47)
	1	8 (47)
	2	1 (6)
**WHO histology, n (%)**		
	A	0
	AB	3 (18)
	B1	0
	B2	7 (14)
	B3	5 (29)
	TC	2 (12)
**Time since first diagnosis (years)**		
	median	0.3
	range	0.1–17.9
**Prior therapy, n (%)**		
	Radiotherapy	1 (6)
	Chemotherapy	3 (18)

ECOG: Eastern Cooperative Oncology Group [37]; WHO histology: World Health Organization classification of tumors of the thymus [6]; TC: thymic carcinoma.

**Table 2:** Clinical characteristics of 17 patients with stage III thymoma after 12 weeks of treatment.

Patient No.	Age [years]	Sex	WHO type	AChR-AB [+/-]	Tumor volume (week 12) [ml]	Tumor volume change (baseline–week 12) [%]	Response (week 12)	Resect-ability^a^ (week 12)
**1**	74	F	B2	-	298	-42	Resp.	res.
**2**	65	F	B3	-	46	-54	Resp.	res.
**3**^**b**^	81	M	B2	+	28	-83	Resp.	not res.
**4**	84	F	AB	+	92	-41	Resp.	not res.
**5**	61	F	AB	-	327	-61	Resp.	not res.
**6**^**b**^	67	F	B3	-	125	-61	Resp.	not res.
**7**	72	F	B3	-	616	-27	Resp.	res.
**8**^**b**^	45	F	TC	-	88	-25	Resp.	not res.
**9**	77	F	B2	-	30	-51	Resp.	not res.
**10**^**b**^	38	F	B2	-	73	-86	Resp.	not res.
**11**	79	F	B3	+	183	-25	Resp.	not res.
**12**^**c**^	49	M	TC	-	-	-	-	-
**13**	71	M	B2	+	21	-55	Resp.	res.
**14**^**c**^	74	F	AB	-	-	-	-	-
**15**	55	M	B2	-	1400	-47	Resp.	not res.
**16**	67	F	B3	+	10	-20	Resp.	res.
**17**	58	F	B2	+/-	100	-77	Resp.	not res.

F: female; M: male; AChR-AB: acetylcholine receptor antibodies (+: titer >0.25 nmol/l); Resp.: Responder; res.: resectable; not res.: not resectable.

^a^ Tumor resectability defined as resectable for radical resection.

^b^ At week 24, tumors of four additional patients (patient no. 3, 6, 8 and 10) were resectable.

^c^ Patient 12 and 14 discontinued before week 12 due to disease progress and adverse events, respectively.

### Treatment Response

**Fig 2:**
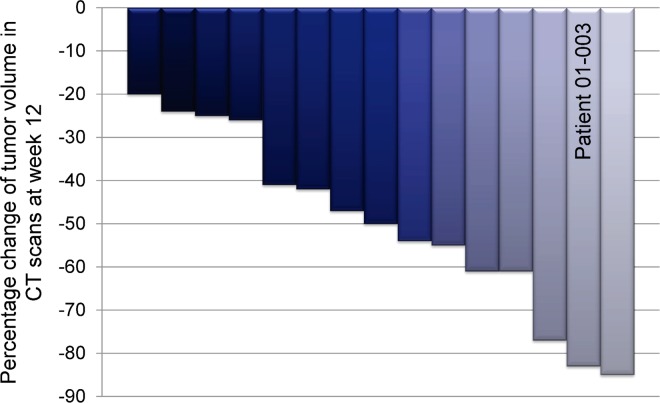
Treatment response to octreotide LAR plus prednisone. Percentage change of tumor volume in CT scans of 15 patients between baseline and week 12 of treatment. Patient number 01–003 shown in Fig 3 is indicated.

Fifteen patients (88%) completed at least 12 weeks of treatment with octreotide LAR plus prednisone. In these patients, the tumor volume decreased by at least 20% (95% CI, 63.6%–98.5%) (Fig 2 and Table 2). Two of the 17 patients recruited (12%) discontinued the study before week 12 due to disease progression (i.e. increased tumor volume at week 6) and occurrence of AEs (one patient each). The patient who showed an increase in tumor volume had a thymic carcinoma.

**Fig 3:**
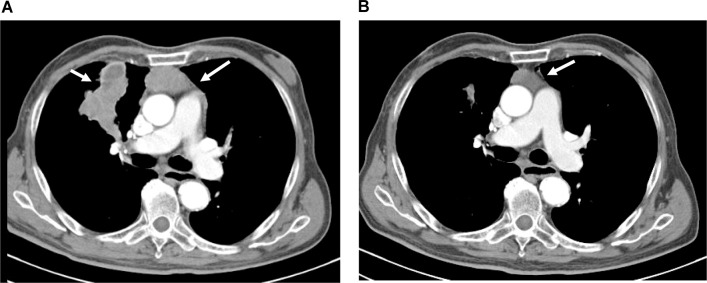
Exemplary CT scans. CT scans of patient number 01–003 were obtained at the beginning of the study (A) and after 12 weeks of treatment with octreotide LAR plus prednisone (B). White arrows indicate tumor lesions.

At baseline, patients had a median tumor volume of 245 ml (range 13–2650 ml). At week 12 of the study, tumor size was reduced by a median of 50% (range: 25%–86%) when compared to baseline in the 15 patients evaluated (Fig 2). The median tumor volume was then 92.1 ml (range 10–1400 ml). Fig 3 exemplarily shows CT scans of one patient (#01–003) at the beginning of the study and after 12 weeks of treatment with octreotide LAR plus prednisone.

At week 12, the tumors of 5 of the 15 patients with tumor response were considered as resectable, and patients successfully underwent radical resection. In these patients, radiological signs of infiltration of adjacent structures were no longer detectable due to tumor regression. The tumors of the remaining 10 patients were still classified as non-resectable after 12 weeks of treatment despite a reduction of tumor mass. Nine of these patients continued the therapy with octreotide LAR and prednisone while one patient immediately discontinued treatment due to AEs. During the following 12 weeks of treatment (week 13–24), two additional patients discontinued due to AEs and one patient died (see section on safety below), whereas six of nine patients continued treatment until week 24. At week 24, the tumors of four additional patients were considered resectable. Compared to baseline, the median reduction of tumor volume at week 24 was 76% in the six patients evaluated. In summary, the overall response rate was 88% for the 17 patients enrolled in our study. All patients presenting with thymoma and completing 12 weeks of treatment responded to therapy. A total of 9 out of 17 patients (53%) reached surgical radical resectability.

After 12 weeks of treatment, no change of myasthenic symptoms was observed when compared to baseline. Although 6 of 17 patients were seropositive for AChR antibodies, none of them showed significant myasthenic symptoms at all visits (Myasthenia gravis score = 0 according to Besinger score [38]). AChR antibodies were detectable in patients with type AB, B2 and B3 thymoma, indicating the presence of a paraneoplastic phenomenon (Table 2). After treatment with octreotide LAR plus prednisone, tumors in four of six AChR sero-positive patients were resectable. The AChR antibody titers continuously decreased during the treatment with octreotide LAR plus prednisone. Antibodies to titin were not detected in any patient.

### Safety

Safety data were consistent with the known safety profile of octreotide LAR and prednisone. A total of 16 of 17 patients (94%) experienced at least one AE. Most AEs were grade 1 or 2; grade 3 AEs occurred in three patients. AEs occurring in more than 12% of patients (≥ 2 patients) are listed in Table 3. The most common AEs were lymphopenia (in 35% of patients), diarrhea (29%), Cushing`s syndrome (24%), and flatulence (24%). The system organ classes in which AEs occurred most frequently were gastrointestinal disorders (12 patients [71%]), followed by infections and infestations (11 patients [65%]) and blood and lymphatic system disorders (7 patients [41%]). One patient died from pulmonary sepsis during the study, which was assessed to be caused by thymoma-associated immunodeficiency during steroid medication and was not suspected to be related to the study medication octreotide LAR.

**Table 3:** Adverse events with a frequency of at least two patients (≥12%).

Adverse event	Any grade N = 17 n (%)	Grade 3 n (%)
**Lymphopenia**	6 (35)	0
**Diarrhea**	5 (29)	0
**Cushing`s syndrome**	4 (24)	0
**Flatulence**	4 (24)	0
**Abdominal pain upper**	3 (18)	0
**Angina pectoris**	3 (18)	0
**Diabetes mellitus**	3 (18)	0
**Infection (Sepsis, Diverticulitis)**	3 (18)	3 (12)
**Leukocytosis**	3 (18)	0
**Pneumonia**	3 (18)	1 (6)
**White blood cell count increased**	3 (18)	0
**Deep vein thrombosis**	2 (12)	0
**Depression**	2 (12)	0
**Lymphocytosis**	2 (12)	0
**Muscle spasms**	2 (12)	0
**Back pain**	2 (12)	0
**Oral candidiasis**	2 (12)	0
**Neutrophilia**	2 (12)	0
**Oral herpes**	2 (12)	0
**Pulmonary embolism**	2 (12)	0
**Visual acuity reduced**	2 (12)	0

Three patients (18%) discontinued treatment due to AEs (deep vein thrombosis, pulmonary embolism, pneumonia, rectal ulcer, sepsis and necrotizing fasciitis). These AEs were judged to be unrelated to octreotide LAR treatment but related to thymoma associated paraneoplastic immunodeficiency (including Good’s syndrome), tumor lysis syndrome, or cortisone treatment.

## Discussion

Here we report the results of a phase II study on octreotide LAR plus prednisone in patients with primary or locally recurrent, unresectable stage III thymic tumors according to the Masaoka staging system. The overall response rate after 3 months of treatment was 88%, with response defined as a reduction of tumor volume of at least 20%. Furthermore, operability was achieved in 29% of patients after 3 months of treatment, in additional 23% of patients after 6 months of treatment (overall operability 52%).

The results of our study confirm the antitumor effects for octreotide LAR plus prednisone that have been observed in two previous phase II trials [35, 36]. In these studies, the treatment of advanced and refractory thymic tumors with somatostatin analogue and prednisone resulted in overall response rates of 30% and 37%. However, in these previous trials, most patients had metastatic thymoma (stage IV) and had undergone previous resection and chemotherapy, which is in contrast to our study. In addition, the different tumor response cut-off of these previous studies (≥50% tumor decrease) might explain why the overall response rates were lower than those observed in our study (30% and 37% vs. 88%). However, the response rate of our cohort was still higher when the same cut-off criteria were applied because 47% of patients showed a tumor reduction of at least 50% (Fig 2 and Table 2). Differences in patient characteristics with respect to prior treatments, resection status, and tumor stage, as well as the use of different octreotide formula (octreotide sc. versus octreotide LAR) might be the reasons for the higher response rate in our study. In line with the latter hypothesis, the LAR formula was demonstrated to be more effective than octreotide sc. in acromegalic patients with response rates of 51% and 37%, respectively [40].

Our results suggest a synergistic effect of octreotide LAR and prednisone. Loehrer et al. [36] evaluated the response of thymic cancers to octreotide monotherapy. In their study, all patients were initially treated by octreotide monotherapy for 2 months. Responding patients continued monotherapy for a maximum of 10 additional months. Partial responses during octreotide monotherapy were then demonstrated in 10% of these patients. Non-responders continued to receive octreotide plus prednisone resulting in an overall response rate of 30% [36]. This response rate corresponds to the response rate found in patients who received the octreotide-prednisone combination therapy throughout the study [35]. In addition, Loehrer et al. [36] observed that patients who were treated with octreotide monotherapy beyond the two initial study months had an objective response later during the study, suggesting that two months of octreotide monotherapy may not have been sufficient for response. However, the mechanisms of the synergistic effect of octreotide LAR and prednisone remain unclear.

Regression of thymoma to glucocorticoid monotherapy has been known for long [41, 42]. Previous reports described a significantly better response in type B1 thymoma than in other thymoma subtypes, presumably due to the high content of glucocorticoid-sensitive immature lymphocytes in type B1 thymomas. However, these studies found apoptotic changes not only in lymphocytes but also in the neoplastic thymic epithelial cells [43, 44]. Furthermore, glucocorticoid receptors were identified in cultured human thymic epithelial cells, and it has been shown that low-dose steroids lead to an increased expression of the somatostatin gene in the thymus [30, 45]. Taking these experimental findings into account, it is tempting to speculate that the favorable response to the combination of octreotide LAR and prednisone in the current study could be due to a synergistic effect of both agents. Further investigations are needed to compare the effect of octreotide LAR with and without prednisone.

Due to the limited number of patients, we cannot draw any conclusion concerning the effects of different WHO histological subtypes and Myasthenia gravis status on response rate. This study did not include patients with the very rare type B1 thymomas that seem to be particularly sensitive to corticosteroid treatment as discussed above. The presence of paraneoplastic Myasthenia gravis did not seem to correlate with tumor growth because no AChR autoantibodies were found in the patients with the largest tumor burden. This is in line with the observation that thymomas with paraneoplastic syndromes are usually detected earlier than those without [46]. AEs were mostly hematologic, gastrointestinal, and infectious or related to the steroid use. This corresponds to the findings of previous studies. Palmieri et al. [35] reported mainly AEs to steroids and gastrointestinal events, whereas Loehrer et al. [36] also observed hematologic and infectious AEs.

We recognize three major limitations in our study. First, the response was defined as the decrease in tumor volume of at least 20%. This does not correspond to the criteria for partial response in solid tumors according to RECIST [46]. However, this assessment is dependent on diameter measurements; generally accepted criteria for volume measurements do not exist [47]. When applying the RECIST criteria of 30% decrease, the overall response rate in our study would be 65%. Second, the limited sample size and, third, the single arm design of the study hamper the generalizability of our results, which, therefore, need confirmatory investigations.

In conclusion, we proved the efficacy of octreotide LAR in combination with prednisone as neoadjuvant treatment for primary or local recurrent unresectable stage III thymic tumors. The AEs were in line with the known safety profile and appear acceptable considering the high response rates. Because of the rarity of thymomas, a randomized multicenter trial will be necessary to confirm our findings. In addition, our results demonstrate that treatment with octreotide LAR in combination with prednisone substantially improves resectability. Finally, if resectability is not achievable in palliative situations, this combination treatment may be an alternative to chemotherapy to maintain stable disease.

## Supporting Information

S1 File Study protocol. (PDF)

S2 File TREND statement checklist1. (JPG)

S3 File TREND statement checklist2. (JPG)

S4 File TREND statement checklist3. (JPG)
