# Supplementary material for: Octreotide LAR and Prednisone as Neoadjuvant Treatment in Patients with Primary or Locally Recurrent Unresectable Thymic Tumors: A Phase II Study
Source: PLoS One. 2016 Dec 16;11(12):e0168215. doi: 10.1371/journal.pone.0168215 (PMC5161359; doi:10.1371/journal.pone.0168215)
Supplement: S1 File — (PDF) [file pone.0168215.s001.pdf]

Clinical Development

Sandostatin® LAR® (octreotide acetate in microspheres for IM injection)

Protocol No. C SMS 995 DE 13

Efficacy of medical treatment with Sandostatin®LAR® in patients with primary inoperable thymoma to reduce tumor size

Author(s): Prof. Berthold Schalke, Prof. Alexander Marx, Birgit Weinkauf, Dr. Christian Hosius

Document type: Clinical Study Protocol

Development Phase: II

Document status: Final

Date: 27. July 2004

Number of pages: 45

Property of Novartis Pharma GmbH
Confidential
May not be used, divulged, published or otherwise disclosed
without the consent of Novartis Pharma GmbH

Signature page for Novartis

Compound name: Sandostatin® LAR®

Study number: C SMS 995 DE 13

Approved by the following:

Dr. Christian Hosius (Clinical Trial Leader)	signature	date
Birgit Weinkauf (Trial Statistician)	signature	date
Prof. Berthold Schalke (Principal Investigator)	signature	date

Signature page for Investigators

Compound name: Sandostatin® LAR®

Protocol number: C SMS 995 DE 13

I have read this protocol and agree to conduct this trial in accordance with all stipulations of the protocol and in accordance with the Declaration of Helsinki.

(Investigator)	signature	date

Table of contents

Signature page for Novartis 2
Signature page for Investigators 3
List of abbreviations 6
1 Introduction 7
1.1 Overview / Pathogenesis of thymomas 7
1.2 State-of-the-Art of Diagnosis and Treatment 7
1.2.1 Histology (WHO Classification) 7
1.2.2 Staging of Thymoma 9
1.2.3 The GETT Staging System 10
1.2.4 Imaging Procedures 11
1.2.5 Traditional Therapy Concepts 12
2 Study objectives 14
3 Investigational plan 15
3.1 Overall study design 15
3.2 Discussion of design 15
3.3 Study population 16
3.3.1 Patient population 16
3.3.2 Inclusion and exclusion criteria 16
3.3.3 Interruption or discontinuation of treatment 17
3.4 Treatment 18
3.4.1 Investigational therapy 18
3.4.2 Patient numbering 19
3.4.3 Blinding 19
3.4.4 Concomitant therapy 19
3.5 Visit schedule and assessments 20
3.5.1 Visit schedule 20
3.5.2 Efficacy assessments 23
3.5.3 Safety assessments 23
3.5.4 Further assessments 26
4 Protocol amendments, other changes in study conduct 26
4.1 Protocol amendments 26
4.2 Other changes in study conduct 27
5 Data management 27
5.1 Data collection 27
5.2 Database management and quality control 27

6 Data analysis ... 28
6.1 Population for analysis ... 28
6.2 Patient demographics / other baseline characteristics ... 28
6.3 Treatments (study drug, concomitant therapies, compliance) ... 28
6.4 Primary objective ... 29
6.4.1 Variable ... 29
6.4.2 Statistical hypothesis, model, and method of analysis ... 29
6.4.3 Handling of missing values/discontinuations ... 29
6.5 Secondary objectives ... 30
6.6 Safety evaluation ... 30
6.7 Sample size and power considerations ... 30
7 References ... 31
8 Procedures and instructions ... 34
8.1 Special safety-related procedures ... 34
8.1.1 Instructions for rapid notification of serious adverse events ... 34
8.1.2 Instructions for completing adverse event case report forms ... 35
8.2 Administrative procedures ... 36
8.2.1 Changes to the protocol ... 36
8.2.2 Monitoring procedures ... 36
8.2.3 Recording of data and retention of documents ... 37
8.2.4 Auditing procedures ... 38
8.2.5 Handling of study medication ... 38
8.2.6 Publication of results ... 38
8.2.7 Disclosure and confidentiality ... 39
8.2.8 Discontinuation of study ... 39
8.3 Ethics and Good Clinical Practice ... 39
8.3.1 Institutional Review Board/Independent Ethics Committee ... 39
8.3.2 Informed consent ... 39
8.3.3 Declaration of Helsinki ... 40
Appendix 1: Reference Pathology ... 41
Appendix 2: Instructions for IM Injection of Sandostatin® LAR® ... 41
Appendix 3: Instructions for ACHR-ab and titin-ab ... 45

List of abbreviations

CRO	Contract Research Organization
DA	disc area
DD	disc diameter
DMB	Data Monitoring Board
DRS	Diabetic Retinopathy Study
ECG	Electrocardiogram
ETDRS	Early Treatment Diabetic Retinopathy Study
HbA_{1c}	glycosylated hemoglobin
IGF-I	insulin-like growth factor-I
IGF BP 1-6	Binding proteins 1-6 of IGF
IVIG	intravenous immuniglobulin G
IVRS	interactive, voice activated randomization system
ITT	intent-to-treat population
LAR	long acting repeatable
MG	Myasthenia gravis
ME	Macular edema
NCR	No carbon required
NPDR	non-proliferative diabetic retinopathy
NVD	new vessels disc
NVE	new vessels elsewhere
PDR	proliferative diabetic retinopathy
PP	Per protocol
VH/PRH	vitreous/preretinal hemorrhage
VEGF	vascular endothelial growth factor

1 Introduction

1.1 Overview / Pathogenesis of thymomas

Thymomas are thymic epithelial tumors and the most frequent neoplasms in the anterior mediastinum. According to the now well accepted WHO histological classification of tumors of the thymus (Rosai, 1999), thymomas are mainly subdivided into WHO type A, AB, B and C thymomas. WHO type C thymomas are equivalent to "thymic carcinomas" that form a histologically and clinically heterogeneous group of a priori malignant thymic epithelial tumors, including thymic neuroendocrine carcinomas (Travis et al, 2994). Though also composed of epithelial cells, germ cell neoplasias of the thymus are not counted among the thymomas.

Tumor stage as defined by the modified Masaoka staging system has been recognized for long as the most important prognostic factor in thymomas and this observation has been confirmed also by the most recent WHO-based studies (Ströbel, JCO 2004).

The diagnosis of thymoma histology by excision or fine needle core biopsy is well established as is the definition of tumor stage by imaging techniques and pathology.

The evaluation of therapeutic strategies in thymomas has been hampered for long by the lack of a generally accepted and biologically meaningful histological thymoma classification. In addition, the definition of "malignancy" in thymoma has been controversial up to now. These problems that can now generally be overcome by the widely accepted WHO classification, that has been shown recently to have independent prognostic value in addition to tumor stage(Ströbel, JCO 2004). Two further problems are more difficult to cope with. First, thymomas are very rare tumors with an incidence of about 1 per million, virtually precluding meaningful studies in single institutions. Second, the majority of the malignant thymomas (roughly 50% of all thymomas) follows a protracted clinical course. Recurrences or metastasis often occur more than 5 years after diagnosis of the primary tumor. As a consequence, comprehensive prospective clinical trials in thymomas have been missing so far (Heijna, 1999), while retrospective analyses suggest that overtreatment of low risk thymomas and undertreatment of high risk thymomas are both common problems in thymoma management. Nevertheless, preliminary adjuvant therapy trials focussing on advanced stage or unresectable thymomas provided valuable clues to the susceptibility of thymomas towards radio- and chemotherapy.

1.2 State-of-the-Art of Diagnosis and Treatment

1.2.1 Histology (WHO Classification)

The histological classification of thymomas is based on the recently published WHO classification (Rosai, 1999; Travis et al., 2004), that is now generally accepted (Table 1). While other thymoma nomenclatures may still be used clinically, the present study requires that all thymomas be "labeled" as type A, AB, B1-3, or C according to WHO-defined criteria. Furthermore, a diagnosis of a WHO type C thymoma requires further subclassification according to histogenesis and tumor grade (see next paragraph).

Table 1. WHO classification of thymic epithelial tumors (Rosai, 1999) as compared to the most widely used traditional classifications, i.e. the clinico-pathological classification (Levien and Rosai, 1976) and the "histogenetic classification" (Marino and Müller-Hermelink, 1986; Müller-Hermelink, 2000). Irrespective of the histological thymoma terminology applied in a given clinical setting, it is obligatory that each thymoma be classified according to the WHO criteria and nomenclature.

Classification of Thymoma

WHO Type	Clinico-pathological Classification (Levine and Rosai, 1976)	Terminology of the "histogenetic classification" for histological thymoma subtypes
A<br>AB	Benign thymoma	Medullary thymoma<br>Mixed thymoma
B1<br>B2<br>B3	Malignant thymomas category I	Predominantly cortical<br>Cortical<br>Well differentiated thymic carcinoma
C	Malignant thymomas category II	Squamous cell carcinoma<br>Basaloid carcinoma<br>Lymphoepithelioma-like carcinoma<br>Sarcomatoid carcinoma (carcinosarcoma)<br>Clear cell carcinoma<br>Mucoepidermoid carcinoma<br>Undifferentiated carcinoma

Each WHO type A, AB, B1, B2 and B3 thymoma subtype defines a quite specific thymoma entity as far as the morphological spectrum and degree of atypia is concerned. These thymoma subtypes occur exclusively in relation to preexisting thymic tissue and exhibit a more or less obvious resemblance to various morphological aspects of the non-neoplastic thymus. Hence, A, AB, and B1-3 thymoma subtypes have been termed "organotypic" thymic tumors.

By contrast, the WHO type C thymoma category comprises tumors that are not unique to the thymus as far as histology is concerned (the genetic basis has yet to be revealed). Type C thymomas form a very heterogenous group of "thymic carcinomas" that exhibit a divers histogenetic spectrum (e.g. squamous cell or adenocarcinoma) and resemble carcinomas outside the thymus. In addition, type C thymomas of a given histogenetic subtype may exhibit a low, moderate or high degree of cytological atypia like extra-thymic carcinomas and should therefore be graded following the rules of general tumor pathology (G1, G2, G3). In summary, labeling a thymic tumor as "WHO type C thymoma" is not sufficient but requires further histogentic classification and (usually) grading to provide a clinically meaningful diagnosis (Table 1).

1.2.2 Staging of Thymoma

Tumor stage according to the modified Masaoka system (Table 2a) as suggested by Shimosato and Mukai (1994) has been shown to be the most significant single prognostic factor for survival in virtually all studies reported in the literature.

Proper staging of thymomas requires:

- imaging studies (see below)
- macroscopic information as provided by the surgeon and pathologist and
- histological evaluation to define invasion beyond an eventual tumor capsule (stage II), infilration into adjacent organs (stage III) or metasasis (stage IV).

Table 2a. Masaoka staging system (1981) modified according to Shimosato and Mukai (1997). The essential modification suggested by Shimosato and Mukai concerns the definition of stage II thymoma: stage II tumors are now defined by infiltration beyond the tumor capsule into the mediastinal fat or thymus. Thymomas infiltrating "into the capsule" are no longer considere as stage II but counted among stage I thymomas.

Staging of Thymoma
(modified Masaoka staging system as proposed by Shimosato and Mukai, 1994/7)

Stage I	Morphologically completely encapsulated and invasion into capsule but not beyond.
Stage II	Microscopic invasion beyond the tumor capsule into surrounding fatty tissue or mediastinal pleura.
Stage III	Microscopic invasion into neighboring organs (i.e. pericardium, great vessels or lung)
Stage IVa	Pleural or pericardial dissemination
Stage IVb	Lymphogenous or hematogenous metastases

1.2.3 The GETT Staging System

The staging system of the Frensh Study Group on Thymic Tumors (GETT) combines both the clinicipathological stage and the results of surgery, i.e. the extent of respectability (Table 2b). Since the degree of surgical resection has been shown by several studies to be of major prognostic significance (see below), it is not surprising that the GETT system has been shown by a couple of authors to be superior to the Masaoka system with respect to prognosis (Guerin, 1988; Gamondes, 1991; Fuentes, 1992; Resbeut, 1995; Cowen, 1995; Mornex, 1995). However, a recent large series from Japan did not find the degree of resection to be a significant independent variable with respect to survival by multivariate analysis when thymomas where classified according to the recent WHO classification and staged according to the Masaoka scheme (Okumura, 2002). By contrast, our recent findings suggest that complete resection (RO= is a major favourable prognostic factor (Ströbel et al., JCO, 2004).

Table 2a. GETT staging system of thymic tumors

**Stage I**	**Encapsulated tumor with or without capsular invasion. Complete resection.**
**Stage II**	**Invasive tumor (beyond capsule). Complete resection.**
**Stage III**	**A. Invasive tumor. Partial resection.**<br>**B. Invasive tumor. Biopsy only.**
**Stage IV**	**A. Supraclavicular or distant pleural invasion**<br>**B. Distant metasasis/metastases**

Histology (WHO)

The diagnosis in any histology report should include one of the "labels" (WHO type A, AB, B1, B2, B3, C) suggested in the WHO classification (Table 13). Since WHO type C thymomas form a very heterogeneous group of carcinomas with widely divergent morphology and clinical presentation (including a wide spectrum of prognostic implications) type C thymomas should be classified further according to the general rules of tumor histopathology (Table 13, 3rd column). While alternative nomenclatures may still be used according to local habits or tradition, all alternative terms should always be complemented by the respective WHO "label" to ensure reproducibility and provide a basis for quality control (reference pathology). Since thymomas frequently exhibit features of two or three (or eventually more) prototypic thymoma subtypes, histology reports should reflect this morphological heterogeneity ("combined thymomas") according to WHO rules (Rosai, 1999; Travis, 2004). Specifically, the approximate percentage of each thymoma component should be given, taking the tumor area of each component into account (based on all available paraffin blocks, see below). The "most malignant" component irrespective of its proportion in a given thymoma is the deciding factor for therapy stratification (see below).

1.2.4 Imaging Procedures

Computed Tomography (CT) and Magnetic Resonance Imaging (MRI)

In myasthenia gravis (MG) patients computed tomography is the standard diagnostic procedure to examine the patient for the occurrence of thymoma vs. thymic hyperplasia, thymic atrophy or thymic lipoma. CT is usually performed with contigous 5mm slice thickness after bolus injection of contrast medium. It is possible to detect correctly size, locallisation, and the correct relationship between thymoma and the contigous structures e.g. mediastinal fat, pericardium, vessles, lung tissue and pleura. Even infiltration into the adjacent organs can be predictet. Magnetic resonance imaging of thymoma or thymic tissue does not have a real advantage compared with CT imaging, only if pericardial infiltration is suspected sagittal MRI seems to be more sensitive than CT. (Hale, 1990; Tregnaghi, 1995; Camera, 1999; Nicolaou, 1996; Emskötter, 1988;)

Functional imaging

Somatostatin receptor scintigraphy (SMS) with 111In-[DTPA-D-Phe1]-octreotid has the potential to visualize thymoma tissue, whereas lyphfollicular thymic hyperplasia, atrophic thymus and thymolipoma fail to accumulate the somatostatin analog peptides. Therefore it is possible to clearly distinguish between tumor and non tumor tissue. Furthermore it is possible to select patients which are positive in the SMS which might benefit from somatostatin neoadjuvant therapy or palliative therapy in advanced tumor stages. (Lastoria, 1999; Marienhagen, 1999;)

1.2.5 Traditional Therapy Concepts

Surgery

Initial surgery aiming at complete resection (RO) is still the mainstay of thymoma therapy and appears to be sufficient treatment in about 50% of (low risk) thymoma patients (Chen, 2002). Incomplete thymoma resection appears to be one of the main adverse prognostic factors (Gamondes, 1991; Fuentes, 1992; Resbeut, 1995; Cowen, 1995; Mornex, 1995; Koh, 1995; Oppitz, 1997; Kohman, 1997; Ströbel, JCO, 2004) though this view has been challenged by some others (Okumura, 2002).

There have been no prospective randomized studies evaluating the various thymectomy procedure applicable in myasthenia gravis patients (Table 3). The same is true for surgery in thymoma patients (with or without myasthenia gravis). However, the trans-sternal approach with removal of the thymoma plus the adjacent non-neoplastic thymus either with or without the mediastinal fat including its cervical extensions has been the standard for years (Jaretzki, 2000). More recently, minimally invasive videoscopic techniques such as video-assisted thoracoscopic thymectomy, VATS (Yim, 1995; Mack, 1996) or video-assisted thoracoscopic extended thymectomy VATET (Scelsi, 1996) have been advocated not only for thymic hyperplasia in MG but also for the removal of encapsulated and invasive thymoma (Roviaro, 2000; Takeo, 2001; Cheng, 2001). However, assessment of efficacy and safety of the minimally invasive techniques require further studies.

Table 3. Classification of thymectomy strategies (Jaretzki, 2000)

T-1	Transcervical Thymectomy<br>a. Basic<br>b. Extended
T-2	Videoscopic Thymectomy<br>a. Classic (VATS, unilateral approach)<br>b. Extended (VATET, bilateral and cervical approach)

T-3	Transsternal Thymectomy<br><br>a. Standard: all visible mediastinal thymus plus cervical thymic extensions<br><br>(resected from below) plus fat adjacent to the thymus<br><br>b. Extended : T-3 a. plus all mediastinal fat
T-4	Transcervical & Transsternal Thymectomy

Somatostatin Receptor Antagonist Treatment

The normal human thymus has been shown to express somatostatin and various somatostatin receptors (SSRs, code-named sst1-5). In some thymomas, somatostatin expression is lost, while the receptors sst1, sst2 and sst3 are present, with sst3 being the main SSR on neoplastic thymic epithelial cells (Ferrone, 2001). Since somatostatin and octreotide exhibit significant growth inhibition of thymoma epithelial cells in vitro, loss of somatostatin production in thymomas has been considered to be of relevance for thymoma oncogenesis (Ferrone, 2001). SSR expression has been the basis for thymoma imaging by [111In-DTPA-D-Phe1] octreotide scintigraphy (Lastoria, 1998; Marienhagen, 1999). In addition, Octreotide was shown to induce complete (Palmieri, 1997; 2001) or partial remission (Lin, 1999) in single cases of advanced thymoma. A recent study treating 16 patients with chemoresistant stage III and IV thymoma and thymic carcinoma with octreotide is promising (Palmieri, 2002) but requires evaluation in a prospective study.

Neoadjuvant Approaches

Neoadjuvant multimodality approaches including preoperative polychemotherapy radiation and octreotide appear promising and worth testing for unresectable thymoma (Shin & Walsh, 1998). Again, small series but no prospective studies have been reported since then (Bretti & Berruti, 2004; Geffen & Bebhorroch, 2001).

Therapy of Myasthenia Gravis

Myasthenia gravis is an autoimmune disease, mediated by autoantibodies directed against the nicotinic AchR at the neuromuscular junction. In 10-20 % of the patients MG is associated with the occurrence of thymomas, in 70-80% with thymitis with lymphofollicular hyperplasia, finally thymic atrophy and thymolipomas are found .

Standard therapy

Symptomatic therapy: The drug of choice is pyridostimine-bromide (e.g. Mestinon^R) . Most patients neeed 5-6 doses/day which range from 30 – 120mg, a dosoage higher than 600mg/day usually does not lead to an increase of therapeutic effect. If the symptomatic therapy is not sufficient to control myasthenic symptoms immunosuppressive therapy has to be started.

Baseline therapy: prednisone: 0.5-3 mg/kg/day. Initially exacerbation of myasthenic symptoms can occur and is not foreseeable, but can be prevented by slowly increasing the dosage. For long term immunosuppression azathioprine (1-3mg/kg/day) Imurek[R]/ Imuran[R] /Azathioprin[R] is the standard drug in the treatment of MG, he dosage has to be adjusted if side effects occur. The effect starts 3-6 months after onset and reaches its maximum at 12 months. (Oosterhuis,1997, Köhler,2003). Other immunosuppressive drugs like cyclophosphamide, methotrexate, cyclosporine and others are also effective but are not used very commonly.

The intravenous use of immunoglobulins (IVIG) can also improve very effective myasthenic symptoms, the usual dosage is 0.4mg/kg/day on 5 consecutive days. Alternatively plasmaexchange (PE) or immunoadorption can be used for eliminating the ACHR antibodies to improve myasthenia gravis in patients which do not respond sufficiently to standard immunosuppression.. The therapeutic effect of IVIG and PE is comparable, but IVIG is easier to apply.

Octreotide Therapy Effects on Immunological and Histological Parameters

Octreotide treatment will be accompanied by 6-weekly evaluation of hematological parameters in the blood, including the number of recent thymic emigrants as defined by a PCR-based TREC detection assay (Buckley & Douek, 2001). In addition, resected thymoma and thymic carcinoma tissue after octreotide treatment will be analyzed for somatostatin receptor expression analysis in situ (IHC, in-situ hybridization, quantitative PT-PCR). In addition, tumor specimens will be studied in comparison with thymomas not treated by octretide using comparative genomic hybridization (CGH), microsatellite analysis (MSA) and Gene Expression Profiling, taking therapy effects into account.

2 Study objectives

Primary objectives

Shrinkage of tumor size and diameter in patients with primary inoperable thymoma in 20 percent (=responder) ·

Secondary objectives

- Shrinkage of tumor size in patients with inoperable thymoma to reach operability.
- Resection status (R0 / R1 /R2) after 3 resp. 6 months of treatment with Sandostatin®LAR®
- histological and flow cytometric changes under treatment with Sandostatin®LAR® : 1) percentage of necrotic area; 2) degree of depletion (none, slight; moderate; marked) of immature T-cells (immunohistochemistry for CD1a, CD99, CD3 expresion; 3) change of T-cell subset composition as revealed by FACS analysis (Ströbel et al. BLOOD, 2002).
- tolerability and safety

3 Investigational plan

3.1 Overall study design

This will be a open label, single arm study of Sandostatin® LAR® treated group, administered i.m. once every 2 weeks in combination with prednison

For this study two stages are planned according to Fleming's one sample multiple testing procedure for phase II clinical trials. If the study cannot be stopped on the first stage after 15 patients due to futility or success, further 10 patients will be recruited, i.e. a maximum of 25 patients in total will be enrolled into this study. A response rate of lower than 20% (≤ 20%) is considered not to warrant further investigation of Sandostatin® LAR® in patients with inoperable thymoma, because this would not be considered as an important advantage as compared to standard treatment. A response rate of ≥ 40% will be considered to warrant further investigation and may result in controlled trials.

If after the first stage not more three responders can be observed, the study will be stopped due to futility. If at least seven responder are observed, the study will be stopped with success. No further patients will be recruited. In case of more than three but lower than seven responder, the second stage with further 10 patients will be performed.

At the second stage the decision with respect to futility or success of the drug will be done based on the total number of responder, i.e. number of responder of 25 patients: futility in case of not more than eight responder, success in case of at least nine responder (for details of Fleming's design see section 6).

Each patient will be treated or observed in the study for 3 month, unless there is an interruption or discontinuation of the study. After 3 month an interims evaluation of operability of the tumor will be performed on the basis of the 6 and 12 week CT examination.

Patients who will reach operability should be treated with study medication till date of surgery. Surgery should be performed at least 4 weeks after end of study visit.

3.2 Discussion of design

Dosage of Sandostatin® LAR®

Sandostatin® LAR® is available in doses of 10, 20 and 30 mg. In both the treatment of acromegaly and carcinoid tumor patients the usual recommended starting dose of Sandostatin® LAR® is 20 mg with an increase in dose to 30 mg in patients who are not adequately controlled with the 30 mg dose. In a double blind, randomized, controlled study of Sandostatin® LAR® in doses of 10, 20 and 30 mg over 6 months in patients with carcinoid tumors (study SMSE 351), there were few statistically significant differences in the individual efficacy parameters between patients treated with the 10, 20 or 30 mg doses of Sandostatin® LAR® once the steady-state octreotide serum concentrations were reached. However, more rapid control of symptoms was achieved with the 20 and 30 mg doses compared with the 10 mg dose without a significant increase in side effects. At steady-state mean (median) octreotide serum concentrations showed an almost linear relationship between doses and octreotide serum concentrations, being 1231 (894) pg/mL for the 10 mg dose, 2620 (2270)

pg/mL for the 20 mg dose and 3928 (3010) pg/mL for the 30 mg dose of Sandostatin® LAR®.

There are data on dose-response relationships with Sandostatin® LAR® in patients with thymoma. In view of the absence of significant safety differences between the three available doses, it is planned to study the maximum available Sandostatin® LAR® dose (30 mg every 2 weeks) in this study to have a better and faster therapeutic effect of the drug. Octreotide was shown to induce complete (Palmieri, 1997; 2001) or partial remission (Lin, 1999) in single cases of advanced thymoma. A recent study treating 16 patients with chemoresistant stage III and IV thymoma and thymic carcinoma with octreotide is promising (Palmieri, 2002) but requires evaluation in a prospective study.

Patients will be previously untreated and shown to be tolerant to a test dose of s.c. Sandostatin® injection prior to treatment start.

A reduction of the dose of Sandostatin to 20 mg is allowed in case of side effects (see also 3.4.5).

3.3 Study population

3.3.1 Patient population

A total number of 25 patients will be included. After recruitment of 15 patients an interim analysis will be performed to evaluate the primary hypothesis.

3.3.2 Inclusion and exclusion criteria

Inclusion criteria

1. Male or female patients aged >18 years

2. Inoperability of thymic tumor. Inoperability is defined as at least adherence of the tumor to the neighbour organs, suspicious to infiltrate neighbour organs so that R0 resection can not be expected.

3. positive result in SMS-szintigraphy,

4. tumor stage: Thymomas of all WHO based histological subtypes (Rosai, 1999; Travis 2004) at **Masaoka stage III** based on histological examination of core biopsies or resection specimens.

5. Patients for whom written informed consent to participate in the study has been obtained

6. Patients with and without thymoma associated paraneoplastic syndrome

7. Demonstrated tolerance to a test dose of s.c. Sandostatin® injection at Visit 1.

Exclusion criteria

1. Performance status 0,1, or 2 (ECOG)

2. Symptomatic cholelithiasis,

3. Pretreatment with Sandostatin®LAR® within the 3 months (?)

4. Patient has received any other investigational agents within 28 days of first day of study drug dosing

5. Patient is < 5 years free of another primary malignancy except: if the other primary malignancy is not currently clinically significant nor requiring active intervention, or if other primary malignancy is a basal cell skin cancer or a cervical carcinoma in situ. Existence of any other malignant disease is not allowed

6. Grade III/IV cardiac problems as defined by the New York Heart Association Criteria. (i.e., congestive heart failure, myocardial infarction within 6 months of study)

7. Severe and/or uncontrolled medical disease (i.e., uncontrolled diabetes, chronic renal disease, or active uncontrolled infection)

8. Liver disease such as cirrhosis, chronic active hepatitis or chronic persistent hepatitis, or persistent ALT, AST, alkaline phosphatase 2 x > upper limit of normal, or total bilirubin 1.5 x > upper limit of normal.

9. Known diagnosis of human immunodeficiency virus (HIV) infection.

10. Abnormal clinical laboratory values considered by the Investigator to be clinically significant and which could affect the interpretation of the study results,

11. Female patients who are pregnant or lactating, or are of childbearing potential and not practicing a medically acceptable method for birth control.

12. Unable to complete the entire study for any reason

3.3.3 Interruption or discontinuation of treatment

The term "discontinuation" refers to a patient's non-completion of the study. In this study information about the discontinuation will be collected during the study.

Patients who, in the opinion of the Investigator, require alternative medical therapy for the thymoma will be discontinued from the study, and the end of study safety evaluations will be completed.

Patients who discontinue from the study prematurely must complete the final safety evaluations at the time of their discontinuation from the study. The Study Completion CRF must be completed for all patients with an explanation of why the patient was withdrawn from the study, even if the patient refused to return for a final visit. Patients who discontinue prematurely due to significant adverse events (AEs) should continue to be followed until resolution of the AE, and the relevant sections of the CRF should be completed as appropriate. Patients who are discontinued due to clinically significant abnormalities in clinical laboratory results should continue to be evaluated until the abnormality resolves or is judged to be permanent.

It will be documented whether or not each patient completed the clinical study. If for any patient either study treatment or observations were discontinued the reason will be recorded. Reasons that a patient may discontinue participation in a clinical study are considered to constitute one of the following:

1. adverse event(s)

2. abnormal laboratory value(s)
3. abnormal test procedure result(s)
4. unsatisfactory therapeutic effect
5. subject's condition no longer requires study treatment
6. protocol violation
7. subject withdrew consent
8. lost to follow-up
9. administrative problems
10. death

Patients who discontinue the study will have their End-of-treatment-visit 4 weeks after the last application of study medication. Discontinued patients can be observed according to the regular schedule.

3.4 Treatment

3.4.1 Investigational therapy

The sponsor will provide study medication kits (Sandostatin® LAR®) to the investigator for each patient randomized to Sandostatin® LAR®. Each medication kit consists of 1 vial of Sandostatin® LAR®, 30 mg, 2 ampoules of vehicle, and 1 injection set containing 1 single use syringe and 2 single use needles (20 G) for intramuscular injection only. Instructions for preparation and administration of the intramuscular injection, by a registered nurse not otherwise involved in the study, are given in **Appendix 2**. It is important to closely follow the mixing instructions given in the appendix. Sandostatin® LAR® must be administered immediately after mixing.

Medication labels will comply with the legal requirements and be printed in the German language. Drug supplies must be kept refrigerated at 2° - 8° C in an appropriate, secure, locked area (e.g. locked refrigerator).

For testing the tolerability to Sandostatin®, each center will be provided with one box of 10 Sandostatin® 100 µg/mL ampoules as a bulk shipment. Each patient will require one ampoule of Sandostatin® injection that will be given s.c. at Visit 1. (See 3.5.3. Special tests)

Somatostatin receptor anatgonist (Octreotide) treatment

Table xy

**1. week**	**2.-12 week**	**week**	
**1.5mg octreotide/day s.c.**	**30mg lanreotide/every 14**	**0,6,12**	**thymomectomy**
**plus 0,6mg**	**days i.m.**	**(18,24)**	

**prednisone/kg/day**	**plus 0,6mg prednisone/kg/day**	**CT-control**	

3.4.2 Patient numbering

Each patient enrolled into the study will be assigned a two digit center number and a two digit patient number. The patient numbers are assigned **sequentially per center**.

3.4.3 Blinding

Not applicable, given the open-label trial design.

3.4.4 Concomitant therapy

All patients will receive Prednison (0,6 mg/kg/d) orally.

No other therapies for thymoma may be administered during the period that the patient is in the study

3.4.4.1 Therapy of Myasthenia gravis

If a patient has at baseline myasthenic symptoms which require symptomatic therapy, the drug of choice is pyridostimine-bromide (e.g. Mestinon[R]) . Most patients can be satisfiable be treated with 5-6 doses/day which usually range from 30 – 120mg, a dosage higher than 600mg/day usually does not show an increase of therapeutic effect. If the symptomatic therapy is not sufficient to control myasthenic symptoms immunosuppressive or immunmodulatory additional to baseline prednisone therapy has to be started.

Baseline therapy: prednisone:0.5-3 mg/kg/day. Initially exacerbation of myasthenic symptoms can occur and is not foreseeable, but can be prevented by slowly increasing the dosage within 1 week up to the final dosage. If myasthenic symptoms are not decreasing after starting cortison therapy it is on the decision of the treating doctor whether the prednisone dose has to to be enhanced. If prednisone therapy for its own is not able to control myasthenic symptoms within 2 weeks or myasthenic crisis impends additional intravenous use of immunoglobulins (IVIG) can also be used to improve myasthenic symptoms. The usual dosage is 0.4mg/kg/day on 5 consecutive days. (Oosterhuis,1997, Köhler,2003).

3.4.4.2 General therapy

The patient may not receive any other somatostatin analogue while in the study. Other investigational agents are prohibited during the course of the study.

Patients may receive, at the discretion of the investigator, appropriate medical and surgical treatment that is not specifically prohibited by this protocol (e.g. they may receive aspirin and/or lipid lowering agents for prevention or treatment of cardiovascular disease and/or angiotensin converting enzyme inhibitors for nephropathy; antidiarrheal agents may be used as needed and nonsteroidal anti-inflammatory drugs may be given to control inflammation at the injection site). If a patient has or develops hypertension, therapy with an angiotensin converting enzyme inhibitor or angiotensin II antagonist is recommended.

3.4.4.3 Treatment of intestinal side effects of study medication

Intestinal side effects may be observed when patients with diabetes will be treated with long acting SMS-analoga. Besides the direct inhibition of secretion this might be caused by a "latent" exocrine insufficiency of the pancreas.

Normally the intestinal side effects will disappear by itself. Additional a combination of enzymes can be administered 1-2 times together with the main courses. (E.g. Pankreon forte ® or any other preparation with similar contents)

A reduction of the dose of Sandostatin to 20 mg is allowed in case of side effects.

3.4.4.4 Treatment compliance

Records of study medication used, dosages administered, and intervals between visits will be kept during the study. Drug accountability will be noted by the field monitor during site visits and at the completion of the trial.

3.5 Visit schedule and assessments

For schedule of study evaluations, see section 3.5.1, Visit schedule.

All female subjects of childbearing potential will have a urine pregnancy test performed at screening. A positive test will exclude the patient from participation in the study.

Development of gallstones has been reported in long-term recipients of both s.c. Sandostatin and Sandostatin® LAR®. Ultrasound examination of the gallbladder before and at about six monthly intervals during Sandostatin® LAR® therapy is, therefore, recommended. If gallstones do occur, they are usually asymptomatic; symptomatic stones should be treated either by dissolution therapy with bile acids or by surgery.

3.5.1 Visit schedule

See next page for visit schedule !

Examination	Scree-ning	Day 1	Week 2	Week 4	Week 6	Week 8	Week 10	Week 12	Week 14	Week 16	Week 18	Week 20	Week 24	EoS
Visit Number	1	2			3			4[1]			5		6	
Informed consent	x													
Medical history	x													
Inclusion/Exclusion criteria	x													
Physical examination	x				X			x			x		x	x
Pregnancy test (urine)	x													
Histology Reference pathology[6]	Before Scree ning													
CT throrax SMS Szintigraphy	Before Scree ning				option al			x			option al		x	x
SMS s.c. test dose	x													
Sandostatin® LAR® injection		x	x	x	X	x	x	x	x	x	x	x	x	x
Prednison daily	x	x	x	x	x	x	x	x	x	x	x	x	x	x
MG-score (Besinger)[3]	x				x			x			x		x	x
Gallbladder Ultrasound	x												x	x
T3, T4, TSH	x													
Biochemistry[4], Hematology[5], ACHR-ab	x[2]				x			x			x		x	x
TEE	x							option al					x	x
Evaluation of								x					x	x

treatment														
Vital signs	x				X			x			x		x	x
ECG	x												x	x
Adverse Events	x	x			X			x			x		x	x
Concomitant Medications	x	x			X			x			x		x	x
Comments	x	x			X			x			x		x	x

1= Visit 4 is End of Study Visit in case of operability

2 = Laborwerte müssen zum Screening nicht wiederholt werden, wenn sie nicht älter als 4 Wochen sind.

3 = MG diagnostic - Repetitive stimuation,, Ache.ab, Titin-ab, MG-Score (Besinger).

4 = Transaminases (AST/SGOT, ALT/SGPT), alkaline phosphatase, total bilirubin, creatinine, urea, glucose, sodium, potassium, calcium, chloride.

5 = Hemoglobin, hematocrit, platelet and leucocyte counts and the differential leucocyte count

6 = Reference pathology will be performed by Prof. Marx/ Prof. Müller-Hermelink, Würzburg

3.5.2 Efficacy assessments

Radiologic assessments (CT)

CT has to be performed with contingous 5mm slice thickness after bolus injection of contrast medium. CT scans will be assessed centrally by PD Dr. Schuierer, Regensburg

Tumor volume will be measured at initial and at follow-up by standard methods. Response is defined as reduction of tumor volume of 20%.

Histologic assessments

The tumours will be assessed centrally by Prof. Marx / Prof. Müller-Hermelink (see appendix 1). Necrosis of tumour tissue will be detected semiquantitatively by planimetry of macroscopic images taken from full specimen sections taken at an intervall of 1 cm (i.e. thickness of slices will be 1 cm). In addition, randomly taken blocks (2 blocks per cm diameter of a given tumor, with each block measuring 1 cm^2) will be stained with H&E and necrotic areas will be quantified by planimetry of low power images taken from each complete section with a digital camera.

Operability/responder status

Operability/responder will be defined as a tumour shrinkage of at, least 20% and is additionally defined by the treating surgeon as no significant adherence of the tumor to the neighbour organs and no longer suspicious to infiltrate neighbour structures

First evaluation of operability after starting the therapy will be performed after 12 weeks, an evaluation of operability of the tumor will be performed on the basis of the 6 and 12 week CT examination.

Patients with a tumour shrinkage of less 20% after 12weeks of octreotide therapy and further infiltration into neighbour organs so that radical resection can not be expected by the treating surgeon can be treated for further 6 or 12 weeks according to the protocol. If after 12 weeks no significant reduction of tumor size occurred or the tumor still infiltrates the adjacent organs the patient is defined as "non responder" The patient can than outside the study protocol either be operated or treated with other chemotherapeutic or radiological therapeutic regimes.Staging according to Masaoka and GETT will be defined by the treating surgeon together with the pathologist referring to histological findings.

3.5.3 Safety assessments

Safety assessments will consist of monitoring and recording all adverse events and serious adverse events, the regular monitoring of hematology and blood chemistry, gall bladder ultrasound examinations at start and at of study, regular measurement of vital signs and the performance of physical examinations.

Adverse events

Information about all adverse events, whether volunteered by the subject, discovered by investigator questioning, or detected through physical examination, laboratory test or other

means, will be collected and recorded on the Adverse Event Case Report Form and followed as appropriate. An adverse event is any undesirable sign, symptom or medical condition occurring after starting study treatment, even if the event is not considered to be treatment-related.

Medical conditions/diseases present before starting study treatment are only considered adverse events if they worsen after starting study treatment. Clinical events occurring before starting study treatment but after signing the informed consent form are recorded on the Medical History/Current Medical Conditions Case Report Form only if the patient receives study treatment. Abnormal laboratory values or test results constitute adverse events only if they induce clinical signs or symptoms or require therapy, when they are recorded on the Adverse Events Case Report Form under the signs, symptoms or diagnosis associated with them.

As far as possible, each adverse event will also be described by:

1. its duration (start and end dates),
2. the severity grade (mild, moderate, severe)
3. its relationship to the study drug (suspected / not suspected),
4. the action(s) taken.

Examples of the severity grade, relationship to study drug and actions taken, as presented in the case report form are provided in Section 8.1.3. "Instructions for completing Adverse Event Case Report Forms".

Serious adverse events

Information about all serious adverse events will be collected and recorded on the Serious Adverse Event Report Form. To ensure patient safety each serious adverse event must also be reported to Novartis within 24 hours of learning of its occurrence. A serious adverse event is defined in general as an untoward (unfavorable) event which:

1. is fatal or life-threatening,
2. required or prolonged hospitalization,
3. was significantly or permanently disabling or incapacitating,
4. constitutes a congenital anomaly or a birth defect,
5. may jeopardize the subject and may require medical or surgical intervention to prevent one of the outcomes listed above

Events not considered to be serious adverse events are hospitalizations occurring under the following circumstances: were planned before entry into the clinical study; are for elective treatment of a condition unrelated to the studied indication or its treatment; occur on an emergency, outpatient basis and do not result in admission (unless fulfilling the criteria above); are part of the normal treatment or monitoring of the studied indication and not associated with any deterioration in condition.

For detailed instructions about completing and returning Serious Adverse Event Report Forms to Novartis refer to Section 8.1.1 Instructions for rapid notification of serious adverse events.

Laboratory evaluations

The following laboratory analyses will be performed by a local laboratory. The following analyses will be performed:

Hematology

Hemoglobin, hematocrit, platelet and leucocyte counts and the differential leucocyte count.

Blood chemistry

Transaminases (AST/SGOT, ALT/SGPT), alkaline phosphatase, total bilirubin, creatinine, urea, glucose, sodium, potassium, calcium, chloride.

Endocrine

T3, T4, TSH

Pregnancy screen

Urine pregnancy test performed at screening.

Sandostatin® s.c. test

At Visit 1 (Week -2), patients will be given a single 100 µg s.c. dose of Sandostatin® (octreotide acetate) injection, to assess tolerability.

ECG evaluation

A standard 12 lead ECG will be performed at screening and at end-of-study-visit. Interpretation of the results will be made by a physician and recorded on the ECG CRF.

Gallbladder ultrasound evaluation

Ultrasound evaluation of the gallbladder and biliary tree will performed at screening and at end-of-study-visit. The results will be recorded on the Gallbladder ultrasound CRF.

Vital signs

Vital sign evaluations will consist of blood pressure, pulse rate, and body weight. The blood pressure will be measured after the patient has been sitting at rest for at least three minutes.

Physical examination

Physical examination including, but not limited to, an examination of: general appearance, skin, neck (including thyroid), eyes, ears, nose, throat, breasts, lungs, heart, abdomen, back, lymph nodes, extremities and nervous system, will be performed. Information about the physical examination must be present in the source documentation at the study site. Significant findings that are present prior to the start of study drug must be included in the Relevant Medical History/Current Medical Conditions Case Report Form. Significant findings made after the start of study drug which meet the definition of an Adverse Event must be recorded on the Adverse Event Case Report Form.

Myasthenia Score

Myasthnia score will be performed according to Besinger.

Gallbladder Ultrasound

A gallbladder ultrasound will be performed at baseline and at end of study.

ECG

A 12 lead electrocardiogram will be performed at baseline and at the end of the study. During the study an ECG need only be performed if, in the investigator's opinion, it is indicated by the patient's symptomatology. Information about ECG findings must be present in the source documentation at the study site. Significant findings that are present prior to the start of study drug must be included in the Relevant Medical History / Current Medical Conditions Case Report Form. Significant findings made after the start of study drug which meet the definition of an Adverse Event must be recorded on the Adverse Event Case Report Form.

3.5.4 Further assessments

Titin-ab

Serum titin-ab will be analyzed by a central laboratory. Blood samples must be obtained prior to dosing with Sandostatin® LAR®. (Please see appendix 3)

ACHR-ab

Serum titin-ab will be analyzed by a central laboratory. Blood samples must be obtained prior to dosing with Sandostatin® LAR®. (Please see appendix 3)

4 Protocol amendments, other changes in study conduct

4.1 Protocol amendments

Any change or addition to this protocol requires a written protocol amendment that must be approved by Novartis and the investigator before implementation. Amendments significantly affecting the safety of subjects, the scope of the investigation or the scientific quality of the study, require additional approval by the IRB/IEC/REB of all centers, and, in some countries, by the regulatory authority. A copy of the written approval of the IRB/IEC/REB, which becomes part of the protocol, must be given to the Novartis monitor. Examples of amendments requiring such approval are:

1. an increase in drug dosage or duration of exposure of subjects
2. a significant change in the study design (e.g. addition or deletion of a control group)
3. an increase in the number of invasive procedures to which subjects are exposed
4. addition or deletion of a test procedure for safety monitoring

These requirements for approval should in no way prevent any immediate action from being taken by the investigator or by Novartis in the interests of preserving the safety of all subjects included in the trial. If an immediate change to the protocol is felt to be necessary by the

investigator and is implemented by him/her for safety reasons Novartis should be notified and the IRB/IEC/REB at the center should be informed within 10 working days.

Amendments affecting only administrative aspects of the study do not require formal protocol amendments or IRB/IEC/REB approval but the IRB/IEC/REB of each center must be kept informed of such administrative changes. Examples of administrative changes not requiring formal protocol amendments and IRB/IEC/REB approval that can be treated as administrative amendments include:

1. changes in the staff used to monitor trials (e.g. Novartis staff versus a CRO)
2. minor changes in the packaging or labeling of study drug

4.2 Other changes in study conduct

Changes in study conduct are not permitted. Any unforeseen changes in study conduct will be recorded in the clinical study report.

5 Data management

5.1 Data collection

Investigators must enter the information required by the protocol onto the Novartis Case Report Forms (CRFs) that are printed on 3-part, no carbon required (NCR) paper. Monitors will review the CRFs for completeness and accuracy, and instruct site personnel to make any required corrections or additions. The CRFs are forwarded to the Data Management of a Novartis nominated Contract Research Organization (CRO) by monitors or by the investigational site, one copy being retained at the investigational site. Once the CRFs are received by the CRO, their receipt is recorded and they are reviewed prior to data entry.

5.2 Database management and quality control

Data items from the CRFs are entered into the study database using double data entry with electronic verification upon second entry. Text items (e.g. comments) are entered once and checked manually against the Case Report Forms..

Subsequently, the information entered into the database is systematically checked by Data Management staff, using error messages printed from validation programs and database listings. Obvious errors will be corrected by the CRO personnel according to the Obvious Correction Document. Other errors or omissions will be entered on Data Query Forms, which will be returned to the investigational site for resolution. A copy of the signed Data Query Form or the original is to be kept with the CRFs at the investigator's site, and once the original or the copy is received at the CRO the resolutions will be entered into the database. The original/copy will be archived with the original CRFs. If necessary a trial specific handling for DQF resolution (e.g. via fax) will be defined in a separate document (e.g. VAP-document) to reduce the time between last DQF written and last DQF in-house to accelerate database lock.

Concomitant medications entered into the database will be coded using a WHODRL Anatomical Therapeutic Chemical dictionary. Coexistent diseases and adverse events will be coded using MEDDRA dictionary.

Safety laboratory data will be performed locally and the results are to be documented in the CRF.

When the database has been declared to be complete and accurate, the database will be locked. Any changes to the database after that time can only be made by joint agreement between the Clinical Trial Leader, the Trial Statistician and the Data Manager.

6 Data analysis

6.1 Population for analysis

Safety and tolerability analyses will be performed on the safety population, which comprises all randomized patients who received at least one dose of randomized medication.

The intent-to-treat population contains all patients of the safety sample for whom at least one post baseline measurement of tumor volume is available. Patients without any post baseline measurement but who nevertheless are to be assessed as treatment failure based on clinical evaluation and/or on obviously fulfilled criteria of increase in tumor volume are to be defined as non-responder. In case of at least one post-baseline value the LOCF procedure will be applied for the measurement at month 3 (primary efficacy parameter).

Confirmatory analysis of the primary efficacy variable and analysis of secondary parameters will be performed for this population.

6.2 Patient demographics / other baseline characteristics

Demographic and baseline characteristics including cancer history will be analyzed descriptively using simple location and dispersion measures for continuous variables and relative and absolute frequencies for categorical variables. Data will be presented for the safety population as well as for the intent-to-treat population.

Medical history will be coded using MedDRA and will be presented by system organ class, MedDRA preferred term and treatment group. Separate tables will be provided for past medical condition and current medical condition.

6.3 Treatments (study drug, concomitant therapies, compliance)

Sandostatin® LAR®

Summary statistics will be presented for the number of injections and for the treatment duration computed as date of last injection minus date of first injection.

Prednison

Summary statistics will be presented for the mean daily dose.

Concomitant Therapies

Concomitant medication will be coded according to WHO Drug Reference List. Summaries will be performed by preferred term and ATC class.

6.4 Primary objective

Primary objective is to show that Sandostatin® LAR® is effective in patients with inoperable thymoma with respect to shrinkage of tumor size and diameter.

6.4.1 Variable

Response is defined as the decrease in tumor volume of 20 % at month 3 as compared to baseline.

6.4.2 Statistical hypothesis, model, and method of analysis

This study is designed according to Fleming's one-sample multiple testing procedure for phase II clinical trials. In total 25 patients will be recruited at two stages, if the trial cannot be stopped before due to futility or success. For this study, a response rate of lower than 0.2 is considered not to warrant further investigation of the drug, whereas a response rate of at least 0.4 is considered to warrant further investigation, i.e. the following hypothesis will be tested:

H_0: $p \leq 0.2$ versus H_1: $p \geq 0.4$

On the first stage 15 patients will be recruited. Based on the number of responder one of the following decisions will be made:

≤ 3 responder →STOP sampling and reject H_1: $p \geq 0.4$
≥ 7 responder →STOP sampling and reject H_0: $p \leq 0.2$
3 < responder < 7 →CONTINUE with further 10 patients

In case of a second stage one of the following decisions will be made based on the number of responder of the total number of patients (i.e. number of responder out of 25 patients):

≤ 8 responder →reject H_1: $p \geq 0.4$
≥ 9 responder →reject H_0: $p \leq 0.2$.

The number of responder will be used for decision making at each stage according to the described rejection and acceptance points.

In addition the relative number of responder will be presented including the 95%-confidence interval.

6.4.3 Handling of missing values/discontinuations

Patients without any post-baseline measurement of tumor volume value will be excluded from analysis unless they are defined as treatment failure based on clinical evaluation and/or obviously fulfilled criteria of increase in tumor volume. In these cases patients are to be defined as non-responder.

If at least one post-baseline value the LOCF procedure will be applied for the measurement at month 3.

6.5 Secondary objectives

Secondary parameters are described in detail in Section 2 and 3. They will be evaluated in an explorative manner.

Operability

A patient's tumor is defined as operable based on the decision of the treating surgeon. In addition the response criteria must be fulfilled.

The number of patients reaching operability will be presented.

Resection status

The frequency distribution of the resection status will be presented based on the categories R0, R1 and ≥ R2.

Histological changes (necrosis)

Necrosis will be assessed as percentage of necrotic tissue. Summary statistics will be presented.

6.6 Safety evaluation

The assessment of safety will be based mainly on the frequency of Adverse Events and on the number of laboratory values that fall outside of pre-determined ranges. Other safety data (e.g. vital signs) will be considered as appropriate.

Adverse Events will be summarized by presenting the number and percentage of patients having any Adverse Event, having an Adverse Event in each body system and having each individual Adverse Event. Any other information collected (e.g. severity or relatedness to study treatment) will be listed as appropriate.

Separate tabulation of adverse events which led to discontinuation of the study, to death, or which were considered serious will also be provided.

Laboratory data will be summarized by presenting summary statistics of raw data and change from baseline values (means, medians, standard deviations, ranges) and by presenting the number and rate of patients with notable abnormalities.

Data from other tests (e.g. electrocardiogram, vital signs and gallbladder ultrasound) will be listed.

6.7 Sample size and power considerations

This study is designed according to Fleming's one-sample multiple testing procedure for phase II clinical trials (Fleming 1982: One-sample multiple testing procedure for phase II clinical trials. Biometrics 38, 143-151). A response rate of lower than 0.2 is considered not to warrant further investigation of the drug, whereas a response rate of at least 0.4 is considered to warrant further investigation, i.e. H0: $p \leq .0.2$ versus H1: $p \geq 0.4$ is to be tested. According to Fleming the following rejection and acceptance points for the null hypothesis will be chosen

for a two stage design with 15 patients at the first stage and additional 10 patient at the second stage:

acceptance point stage 1 (based on 15 patients): 3
acceptance point stage 2 (based on 25 patients): 8
rejection point at stage 1 (based on 15 patients): 7
rejection point at stage 2 (based on 25 patients): 9

In total 25 patients will be enrolled if the study cannot be stopped after the first stage.

This design provides the alpha to be 0.047 and the power to be ≥ 90% if the true rate is 0.50.

7 References

Bretti S, Berruti A, Loddo C et al. (2004)Multimodal management of stages III-Iva malignant thymomas. (2004) Lung Cancer 44(1) : 69-77

Buckley C, Douek D, Newsom Davis J, Willcox N (2001) Mature, long lived CD4+ and CD8+ T cells are generated by thymoma in myasthenia gravis. Ann Neurol 50:64-72

Chen G, Marx A, Wen-Hu C,Yong J, Puppe B, Stroebel P, Müller-hermelink HK (2002) New WHO Histlogic Classification predicts prognosis of thymic epithelial tumors . Cancer 95:420-429

Emskötter T, Trampe H, Lachenmayer L (1988) Kernspintomographie bei Myasthenia gravis. Eine Alternative zur mediastinalen Computertommographie. Dtsch Med Wochenschr, 113, 1508-1510

Gamondes JP, Balawi A, Greenland T et.al.,(1991) Seventeen years of surgical treatment of thymoma: factors influencing survival. Eur J Cardothorac Surg., 5(3) :124-131

Geffen DB, Benharroch D, Yellin A et al. (2001) Multimodal tratment mo metastatic thymic carcinoma including high dose chemotherapy with autologous stem cell transplantation: report of a case with more than 4-year disease free survival. Am J Clin Oncol 24(6): 566-569

Hejna M, Haberl I, Raderer M,(1999) Nonsurgical management of malignat thymoma. Cancer 85:1871-1884

Jaretzki A 3rd, Barohn RJ, Ernstoff RM et al.(2000) Myasthenia gravis: recommendations for clinical research standards. Ann Thorac Surg 70(1): 327-334

Köhler W, Sieb JP, Myasthenia gravis, Uni Med, Bremen,London , Boston , 88-119, 2003

Lastoria S, Vergara E, Calmieri G, Acampa W, Varella P, Caraco C et al..(1998) In vivo detection of malignant thymic masses by Indium-111DTPA-D-Phe1-octreotide scintigraphy. J Nucl Med. 39,634-639

Levine GD, Rosai J (1978) Thymic hyperplasia and neoplasia: a review of current concepts. Hum Pathol. 9:495-515

Loehrer PJ Sr, Wang W, Johnson DH, Aisner SC, Ettinger DS, Eastern Cooperative Oncology Group Phase II Trial, Octreotide alone or with prednisone in patients with advanced thymoma and thymic carcinoma: an Eastern Cooperative Oncology Group Phase II Trial. J Clin Oncol. 2004 Jan 15;22(2):293-9. Erratum in: J Clin Oncol. 2004 Jun 1;22(11):2261.

Marienhagen J, Schalke B, Aebert H, Held P, Eilles C, Bogdahn U (1999) Somatostatin receptor scintigraphy in thymoma imaging method and clinical application. Pathol Res Pract, 195, 575-581

Marino M, Müller-Hermelink HK (1985) Thymoma and thymic carcinoma. Relation of thymoma epithelial cells to the cortical and medullary differentiation of thymus. Virchows Arch A Pathol Anat Histopathol. 407(2):119-149

Masaoka A, Monden Y, Nakahara K,Tanioka T, Follow up study of thymomas with special reference to their clinical stages.(1981) Cancer. 48:2485 -2492

Mornex F, Resbeut M, Richaud P et. Al., (1995) Radiotherapy and chemotherapy for invasive thymomas: amulticentric retrospective review of 90 cases. The FNCLCC trialists. Federation Nationale des Centres de Lutte Contre le Caancer. Int J Radiat Oncol Biol Phys 33(2):545

Müller- Hermelink HK, Marx A. (2000) Thymoma. Curr Opin Oncol. 12: 426-433

Nicolaou S, Müller NL, Li DKB, Oger JJF, (1996)0 Thymus in myasthenia gravis: comparison of CT and pathologic findings and clinical outcome after Thymectomy. Radiology, 201, 471-474

Okomura M, Ohta M, Tateyama H et al,(2002) The world Health Organisation histologic classification system reflects the oncologic behavior of thymoma: a clinical study of 273 patients Cancer 94:624-632

Oosterhuis HJGH, Myasthenia gravis, Groningen Neurological Press pp 68-92,1997

Palmieri G, Lastoria S, Colao A, Vergara E, Varella P, Biondi E, Selleri C, Catalano L, Lombardi G, Bianco AR, Salvatore M (1997) Succesfull treatment of a patient with a thymoma and pure red cell aplasia with octreotide and prednisone. N Engl J Med 336(14): 1039

Palmieri G, Montella L, Lastoria S (2001) Thymoma and somastostatin analogs: Biology, diagnostic and clinical practice. 26(3): 193-195

Palmieri G, Montella L, Martignetti A, Muto P, Di Vizio D, De Chiara A, Lastoria S (2002) Cancer 94(5): 1414-1420

Resbeut M, Mornex F, Richaud P et. al. (1995) Radiotherapy of thymoma. Study of the literatureapropos of retrospectice and multicenter series of 149 cases. Bull Cancer Radiother.

Rosai J (1999),Histological typing of tumors of the thymus. (1999) Berlin:Springer Verlag

Shimosato Y, Mukai K,(1997) Tumors of the mediastininum. In: Rosai J, Sobin LH, editors. Atlas of tumor pathology. 3rd ed. Washington, DC: Armed Forces Institute of Pathology: 93, 93-115

Shin DM, Walsh GL, Komaki R et.al.(1998) A mulidisciplinary approach to therapy for unresectable malignant thymoma. Ann Intern Med. 129(2) 100-104

Tregnagh A, Decandia A, Calderone M (1995) Imaging of the Thymus gland in myasthenia gravis. Radiol Med (Torino), 404-409

Woltering EA et al. Somatostatin analogs: angiogenesis inhibitors with novel mechanisms of action. Investigational New Drugs 15: 77-86, 1997.

8 Procedures and instructions

8.1 Special safety-related procedures

8.1.1 Instructions for rapid notification of serious adverse events

Reporting responsibility

Each serious adverse event must be reported by the investigator to Novartis or to the relevant Contract Research Organization within 24 hours of learning of its occurrence, even if it is not felt to be treatment-related. Follow-up information about a previously reported serious adverse event must also be reported within 24 hours of the investigator receiving it. If the serious adverse event is not previously documented (new occurrence) and is thought to be related to the Novartis study drug (or therapy), a Clinical Safety & Epidemiology Department associate may urgently require further information from the investigator for Health Authority reporting. Novartis may need to issue an investigator notification, to inform all investigators involved in any study with the same drug (or therapy) that this serious adverse event has been reported. Any serious adverse event occurring in a patient after providing informed consent, whilst receiving study treatment and until 1 month after stopping it must be reported.

Reporting procedures

The investigator must complete the Serious Adverse Event Report Form in English, assess the relationship to study treatment and send the completed, signed form by fax within 24 hours to the local Novartis Clinical Safety & Epidemiology Department (for trials monitored by Novartis) OR to the relevant Contract Research Organization. The Contract Research Organization, after ensuring that the form is accurately and fully completed, must then fax it to the Clinical Safety & Epidemiology Department within 24 hours of receipt. The original copy of the Serious Adverse Event Form and the fax confirmation sheet must be kept with the case report form documentation at the study site

Follow-up information is sent to the same person sent the original Serious Adverse Event Form. A new serious adverse event form is sent, stating that this is a follow-up to the previously reported serious adverse event and giving the date of the original report. Each re-occurrence, complication or progression of the original event should be reported as a follow-up to that event. The follow-up information should describe whether the event has resolved or continues, if and how it was treated, and whether the patient continued or discontinued study participation. The form and fax confirmation sheet must be retained. Refer to the Novartis guidelines as needed for instructions for completing the Serious Adverse Event Form.

Contact persons and numbers

The telephone and telefax numbers of the local Clinical Research (CR) contact person, the contact person at the Clinical Research Organization (CRO) and the contact person in the local department of Clinical Safety and Epidemiology (CS&E), specific to the site, are all listed in the investigator folder provided for each individual site.

8.1.2 Instructions for completing adverse event case report forms

Each adverse event is to be reported on an Adverse Event Case Report Form. As far as possible, each adverse event must also be described by:

1. its duration (start and end dates),
2. its severity grade (mild, moderate, severe),
3. its relationship to the study drug (suspected / not suspected),
4. the action(s) taken

Examples of the severity grade, relationship to study treatment and actions taken, as presented in the case report form, are provided below.

The severity grade of an adverse event provides a qualitative assessment of the extent or intensity of an adverse event, as determined by the investigator or as reported by the subject. The severity grade does not reflect the clinical seriousness of the event, only the degree or extent of the affliction or occurrence (e.g. severe nausea, mild seizure), and does not reflect the relationship to study drug.

Severity grade for an adverse event

1 = Mild	the degree/extent/intensity of the event is **mild**
2 = Moderate	the degree/extent/intensity of the event is **moderate**
3 = Severe	the degree/extent/intensity of the event is **severe**

The relationship between the administration of study drug and the occurrence of the adverse event is described as belonging to one of only 2 categories, either suspected by the investigator or not suspected by the investigator.

Relationship of adverse events to study drug

0 = Not suspected	The temporal relationship of the clinical event to study drug administration makes **a causal relationship unlikely**, or other drugs, therapeutic interventions or underlying conditions provide a sufficient explanation for the observed event.
1 = Suspected	The temporal relationship of the clinical event to study drug administration makes a **causal relationship possible**, and other drugs, therapeutic interventions or underlying conditions do not provide a sufficient explanation for the observed event.

The actions taken in response to an adverse event are described on a numerical scale, from 0 to 5 that cover the various possibilities. One or more of these is to be selected.

Actions taken in response to an adverse event

0 = No action taken
1 = Study drug dosage adjusted / temporarily interrupted
2 = Study drug permanently discontinued due to this adverse event
3 = Concomitant medication taken
4 = Non-drug therapy given
5 = Hospitalization / prolonged hospitalization

8.2 Administrative procedures

8.2.1 Changes to the protocol

Any change or addition to this protocol requires a written protocol amendment that must be approved by Novartis and the investigator before implementation. Amendments significantly affecting the safety of subjects, the scope of the investigation or the scientific quality of the study, require additional approval by the IRB/IEC of all centers, and, in some countries, by the regulatory authority. A copy of the written approval of the IRB/IEC, which becomes part of the protocol, must be given to the Novartis monitor. Examples of amendments requiring such approval are:

1. an increase in drug dosage or duration of exposure of subjects
2. a significant change in the study design (e.g. addition or deletion of a control group)
3. an increase in the number of invasive procedures to which subjects are exposed
4. addition or deletion of a test procedure for safety monitoring

These requirements for approval should in no way prevent any immediate action from being taken by the investigator or by Novartis in the interests of preserving the safety of all subjects included in the trial. If an immediate change to the protocol is felt to be necessary by the investigator and is implemented by him/her for safety reasons Novartis should be notified and the IRB/IEC at the center should be informed within 10 working days.

Amendments affecting only administrative aspects of the study do not require formal protocol amendments or IRB/IEC approval but the IRB/IEC of each center must be kept informed of such administrative changes. Examples of administrative changes not requiring formal protocol amendments and IRB/IEC approval that can be treated as administrative amendments include:

1. changes in the staff used to monitor trials (e.g. Novartis staff versus a CRO)
2. minor changes in the packaging or labeling of study drug

8.2.2 Monitoring procedures

Before study initiation, at a site initiation visit or at an investigator's meeting, a Novartis representative will review the protocol and case report forms (CRFs) with the investigators and their staff. During the study the Novartis monitor will visit the site regularly, to check the completeness of patient records, the accuracy of entries on the CRFs, the adherence to the protocol and to Good Clinical Practice, the progress of enrollment, and also to ensure that study medication is being stored, dispensed and accounted for according to specifications.

The investigator and key trial personnel must be available to assist the Novartis monitor during these visits.

The investigator must give the monitor access to relevant hospital or clinical records, to confirm their consistency with the CRF entries. No information in these records about the identity of the subjects will leave the study center. Novartis monitoring standards require full verification for the presence of informed consent, adherence to the inclusion/exclusion criteria, documentation of SAEs and the recording of primary efficacy and safety variables. Additional checks of the consistency of the source data with the CRFs are performed according to the study-specific monitoring plan.

The investigator is responsible for completing the CRFs within 5 days of the patient's visit and the Novartis monitor is responsible for reviewing them and clarifying and resolving any data queries. The completed and corrected CRFs for completed visits will be collected by the Novartis monitor initially, and may then be either collected or sent for data processing, as arranged by the Novartis monitor. A copy of the CRFs is retained by the investigator, who must ensure that it is stored with other study documents, such as the protocol, the investigators brochure and any protocol amendments, in a secure place.

8.2.3 Recording of data and retention of documents

Data on subjects collected on CRFs during the trial will be documented in an anonymous fashion and the subject will only be identified by the subject number, and by his/her initials if also required. If, as an exception, it is necessary for safety or regulatory reasons to identify the subject, both Novartis and the investigator are bound to keep this information confidential.

All the information required by the protocol should be provided and any omissions require explanation. All CRFs must be completed and available for collection no more than 5 days after the patient's visit, so that the monitor may check the entries for completeness, accuracy and legibility, ensure the CRF is signed by the investigator and transmit the data to Novartis.

All entries to the CRFs must be made clearly in black ball-point pen, to ensure the legibility of self-copying or photocopied pages. Corrections are made by placing a single horizontal line through the incorrect entry, so that it can still be seen, and placing the revised entry beside it. The revised entry must be initialed and dated by a member of the investigator's research team authorized to make CRF entries. Correction fluid must not be used.

The investigator must maintain source documents for each patient in the study. All information on CRFs must be traceable to these source documents, which are generally maintained in the patient's file. The source documents should contain all demographic and medical information, including laboratory data, electrocardiograms, etc., also a copy of the signed informed consent form, which should indicate the study number and title of the trial.

Essential documents, as listed below, must be retained by the investigator for as long as needed to comply with national and international regulations (generally 2 years after discontinuing clinical development or after the last marketing approval). Novartis will notify the investigator(s)/institution(s) when the study-related records are no longer required. The investigator agrees to adhere to the document retention procedures by signing the protocol. Essential documents include:

1. IRB/IEC approvals for the study protocol and all amendments

2. all source documents and laboratory records
3. CRF copies
4. patients' informed consent forms
5. FDA form 1572
6. any other pertinent study document

8.2.4 Auditing procedures

In addition to the routine monitoring procedures, a Good Clinical Practice Quality Assurance Unit exists within Novartis. This unit conducts audits of clinical research activities in accordance with internal SOPs to evaluate compliance with the principles of Good Clinical Practice. A regulatory authority may also wish to conduct an inspection (during the study or even after its completion). If an inspection is requested by a regulatory authority, the investigator must inform Novartis immediately that this request has been made.

8.2.5 Handling of study medication

All study medication will be supplied to the principal investigator by Novartis. Drug supplies must be kept in an appropriate, secure area (e.g. locked cabinet) and stored in accordance with the conditions specified on the drug labels. The investigator must maintain an accurate record of the shipment and dispensing of the study drug in a drug accountability ledger, a copy of which must be given to Novartis at the end of the study. An accurate record of the date and amount of study drug dispensed to each subject must be available for inspection at any time.

All drug supplies are to be used only for this protocol and not for any other purpose. The investigator must not destroy any drug labels, or any partly-used or unused drug supply. At the conclusion of the study, and, as appropriate during the course of the study, the investigator will return all used and unused drug containers, drug labels and a copy of the completed drug disposition form to the Novartis monitor or to the Novartis address provided in the investigator folder provided for each site.

8.2.6 Publication of results

Any formal presentation or publication of data collected from this trial will be considered as a joint publication by the investigator(s) and the appropriate personnel of Novartis. Authorship will be determined by mutual agreement. For multicentre studies, it is mandatory that the first publication is based on data from all centers, analyzed as stipulated in the protocol by Novartis statisticians, and not by the investigators themselves. Investigators participating in multicentre studies agree not to present data gathered from one center or a small group of centers before the full, initial publication, unless formally agreed to by all other investigators and Novartis.

Novartis must receive copies of any intended communication in advance of publication (at least 15 working days for an abstract or oral presentation and 45 working days for a journal submission). Novartis will review the communications for accuracy (thus avoiding potential discrepancies with submissions to health authorities), verify that confidential information is not being inadvertently divulged and to provide any relevant supplementary information.

Authorship of communications arising from pooled data will include members of each of the contributing centers as well as Novartis personnel.

8.2.7 Disclosure and confidentiality

By signing the protocol, the investigator agrees to keep all information provided by Novartis in strict confidence and to request similar confidentiality from his/her staff and the IRB/EC. Study documents provided by Novartis (protocols, investigators' brochures, CRFs and other material) will be stored appropriately to ensure their confidentiality. The information provided by Novartis to the investigator may not be disclosed to others without direct written authorization from Novartis, except to the extent necessary to obtain informed consent from patients who wish to participate in the trial.

8.2.8 Discontinuation of study

Novartis reserves the right to discontinue any study for administrative reasons at any time. If appropriate, reimbursement for reasonable expenses will be made.

8.3 Ethics and Good Clinical Practice

This study must be carried out in compliance with the protocol and in accordance with Novartis standard operating procedures. These are designed to ensure adherence to Good Clinical Practice, as described in the following documents:

1. ICH Harmonized Tripartite Guidelines for Good Clinical Practice 1996.
2. Directive 91/507/EEC, the Rules Governing Medicinal Products in the European Community.
3. Declaration of Helsinki, concerning medical research in humans (Recommendations Guiding Physicians in Biomedical Research Involving Human Subjects, Helsinki 1964, amended Tokyo 1975, Venice 1983, Hong Kong 1989, Somerset West 1996).

The investigator agrees, when signing the protocol, to adhere to the instructions and procedures described in it and thereby to adhere to the principles of Good Clinical Practice that it conforms to.

8.3.1 Institutional Review Board/Independent Ethics Committee

Before implementing this study, the protocol, the proposed informed consent form and other information to subjects, must be reviewed by a properly constituted Institutional Review Board/Independent Ethics Committee (IRB/IEC). A signed and dated statement that the protocol and informed consent have been approved by the IRB/IEC must be given to Novartis before study initiation. The name and occupation of the chairman and the members of the IRB/IEC must be supplied to Novartis. Any amendments to the protocol, other than administrative ones, must be approved by this committee.

8.3.2 Informed consent

The investigator must explain to each subject (or legally authorized representative) the nature of the study, its purpose, the procedures involved, the expected duration, the potential risks and benefits involved and any discomfort it may entail. Each subject must be informed that

participation in the study is voluntary and that he/she may withdraw from the study at any time and that withdrawal of consent will not affect his/her subsequent medical treatment or relationship with the treating physician.

This informed consent should be given by means of a standard written statement, written in non-technical language. The subject should read and consider the statement before signing and dating it, and should be given a copy of the signed document. If written consent is not possible, oral consent can be obtained if witnessed by a signed statement from one or more persons not involved in the study, mentioning why the patient was unable to sign the form. No patient can enter the study before his/her informed consent has been obtained.

The informed consent form is part of the protocol, and must be submitted by the investigator with it for IRB/IEC approval. Novartis supplies a proposed informed consent form, which complies to regulatory requirements and is considered appropriate for the study. Any changes to the proposed consent form suggested by the Investigator must be agreed to by Novartis before submission to the IRB/IEC and a copy of the approved version must be provided to the Novartis monitor after IRB/IEC approval.

8.3.3 Declaration of Helsinki

The investigator must conduct the trial in accordance with the principles of the Declaration of Helsinki. Copies of the Declaration of Helsinki and amendments will be provided upon request or can be accessed via the website of the World Medical Association at http://www.wma.net/e/policy/17-c_e.html.

Appendix 1: Reference Pathology

1. Core needle biopsies (at least 1 cm long) should be fixed as soon as possible in buffered (neutral) 4% formalin and sent to:

Prof. Dr. A. Marx

Pathologisches Institut

Universität Würzburg

Josef-Schneider-Str. 2

97080 Würzburg

(Tel.: (+49) (0)931 201-47421 or -47420).

Histological evaluation of H&E stained sections is obligatory and may eventually be complemeted by special stains (Giemsa, Gomöri, PAS) and immunohistochemistry.

2. **Resections specimens** should preferably be sent unfixed and sterile on water ice at 0°C (to prevent freezing !) to Prof. Marx, Würzburg, as soon as possible (ICE courier is prefered). Announcement of the material by phone is kindly requested to ensure proper preservation and fast processing of the material on arrival in Würzburg: Tel: (+49) (0)931 201-47421 or –47420.

In case that transportation of unfixed material on ice at 0oC is not feasible, it would be highly desirable that the local pathologists preserves fresh and unfixed tumor material (~ 1cm^3 of vital tumor) in a –70°C deep freezer after snap freezing in liquid nitrogen. Resection specimen that can not be sent on ice immediately (i.e. the same day) should be fixed in buffered (neutral) 4% formalin and sent to Prof. Marx by overnight mail or courier.

Appendix 2: Instructions for IM Injection of Sandostatin® LAR®

FOR DEEP INTRAGLUTEAL INJECTION ONLY

Hinweise zur intraglutäalen Injektion von Sandostatin LAR-Monatsdepot:

Inhalt der Packung:

Befolgen Sie sorgfältig die nachfolgenden Anweisungen, um eine vollständige Benetzung des Pulvers und seine gleichmäßige Suspension vor der i.m. Injektion zu gewährleisten.

Die Suspension mit Sandostatin LAR-Monatsdepot darf erst unmittelbar vor der Anwendung hergestellt werden.

Sandostatin LAR-Monatsdepot darf nur durch entsprechend geschultes medizinisches Fachpersonal angewendet werden.

Die Durchstechflasche mit Sandostatin LAR-Monatsdepot und die Fertigspritze mit dem Suspensionsmittel sollten vor der Anwendung auf Raumtemperatur gebracht werden.

Entfernen Sie die Schutzkappe von der Durchstechflasche mit Sandostatin LAR-Monatsdepot. Stellen Sie durch leichtes Klopfen an die Durchstechflasche sicher, dass sich das Pulver am Boden der Durchstechflasche befindet.

Entfernen Sie die Schutzkappe von der Fertigspritze mit dem Suspensionsmittel.

Befestigen Sie eine der mitgelieferten Nadeln auf der Fertigspritze mit dem Suspensionsmittel.

Desinfizieren Sie den Gummistopfen der Durchstechflasche mit einem Alkoholtupfer.

Stechen Sie die Nadel der Fertigspritze durch die Mitte des Gummistopfens der Durchstechflasche mit dem Sandostatin LAR-Monatsdepot.

Das gesamte Suspensionsmittel wird dann behutsam in die Durchstechflasche injiziert, indem man die Flüssigkeit langsam auf der Innenseite der Durchstechflasche herabfließen lässt. Das Suspensionsmittel darf nicht direkt auf das Pulver injiziert werden. Ziehen Sie anschließend die Nadel aus der Durchstechflasche.

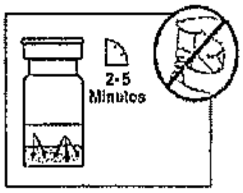

Die Durchstechflasche nicht bewegen, bis das Suspensionsmittel das Pulver mit Sandostatin LAR-Monatsdepot vollständig befeuchtet hat (mindestens 2 – 5 Minuten). Überprüfen Sie diesen Vorgang, **ohne die Durchstechflasche umzudrehen**. Wenn trockene Pulverstellen vorhanden sind, lassen Sie den Vorgang des Benetzens selbstständig weiterlaufen.

Sie können jetzt den Patienten für die Injektion vorbereiten.

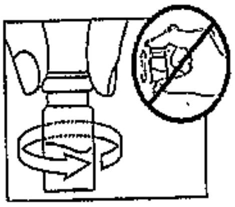

Sobald das Pulver vollständig benetzt ist, sollte die Durchstechflasche 30 – 60 Sekunden vorsichtig gedreht werden, bis eine homogene, milchige Suspension entsteht. **Starkes Schütteln ist zu vermeiden.** Dadurch könnte die Suspension ausflocken und damit unbrauchbar werden.

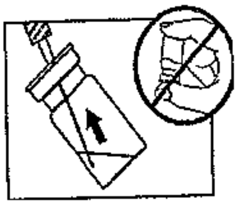

Unmittelbar danach die Nadel der Fertigspritze erneut durch den Gummistopfen stechen. Dann mit nach unten gehaltener Nadelspitze aus der im ca. 45° Winkel schräg gehaltenen Durchstechflasche die gesamte Suspension langsam in die Spritze aufziehen. Während dieses Vorgangs **die Durchstechflasche nicht umdrehen**, weil dies die Menge der entnommenen Suspension beeinflussen könnte. Üblicherweise verbleibt ein kleiner Rest der Suspension an den Wänden oder am Boden der Durchstechflasche. Dies wird durch eine Überfüllung berücksichtigt.

Unmittelbar die Nadel wechseln (zweite mitgelieferte Nadel)

Die Anwendung muss unmittelbar nach der Herstellung der Suspension erfolgen. Falls erforderlich, die Spritze mehrfach behutsam wenden, um die Suspension homogen zu halten. Spritze luftleer machen.

Die Injektionsstelle mit einem Alkoholtupfer desinfizieren. Die Nadel in den rechten oder linken Gesäßmuskel stechen und den Kolben etwas zurückziehen, um sicherzustellen, dass kein Blutgefäß getroffen wurde. Danach die homogene Suspension unter stetigem Druck sofort durch eine langsame, tiefe intraglutäale Injektion intramuskulär injizieren. Wenn die Nadel verstopft ist, ist eine neue Nadel mit dem selben Durchmesser (1,1 mm; 19 Gauge) zu verwenden.

Sandostatin LAR-Monatsdepot darf nur über eine tiefe intraglutäale Injektion und niemals i.v. verabreicht werden. Wenn ein Blutgefäß getroffen wurde, ist eine neue Nadel zu verwenden und eine andere Injektionsstelle zu wählen.

Sandostatin® LAR® must be given only by deep intragluteal injection, never i.v. If a blood vessel has been penetrated, select another injection site.

Appendix 3: Instructions for ACHR-ab and titin-ab

- 10 ml blood should be drawn and centrifuged immediately for 10 min at 4000Xg
- ml of serum should be divided into 2 aliquots of 2.5 ml
- The serum should be stored at -80°C.
- Storage at -20°C is possible only for 6 weeks.
- For posting please see Investigators File.
