# Supplementary figures and images for: Octreotide LAR and Prednisone as Neoadjuvant Treatment in Patients with Primary or Locally Recurrent Unresectable Thymic Tumors: A Phase II Study
Source: PLoS One. 2016 Dec 16;11(12):e0168215. doi: 10.1371/journal.pone.0168215 (PMC5161359; doi:10.1371/journal.pone.0168215)

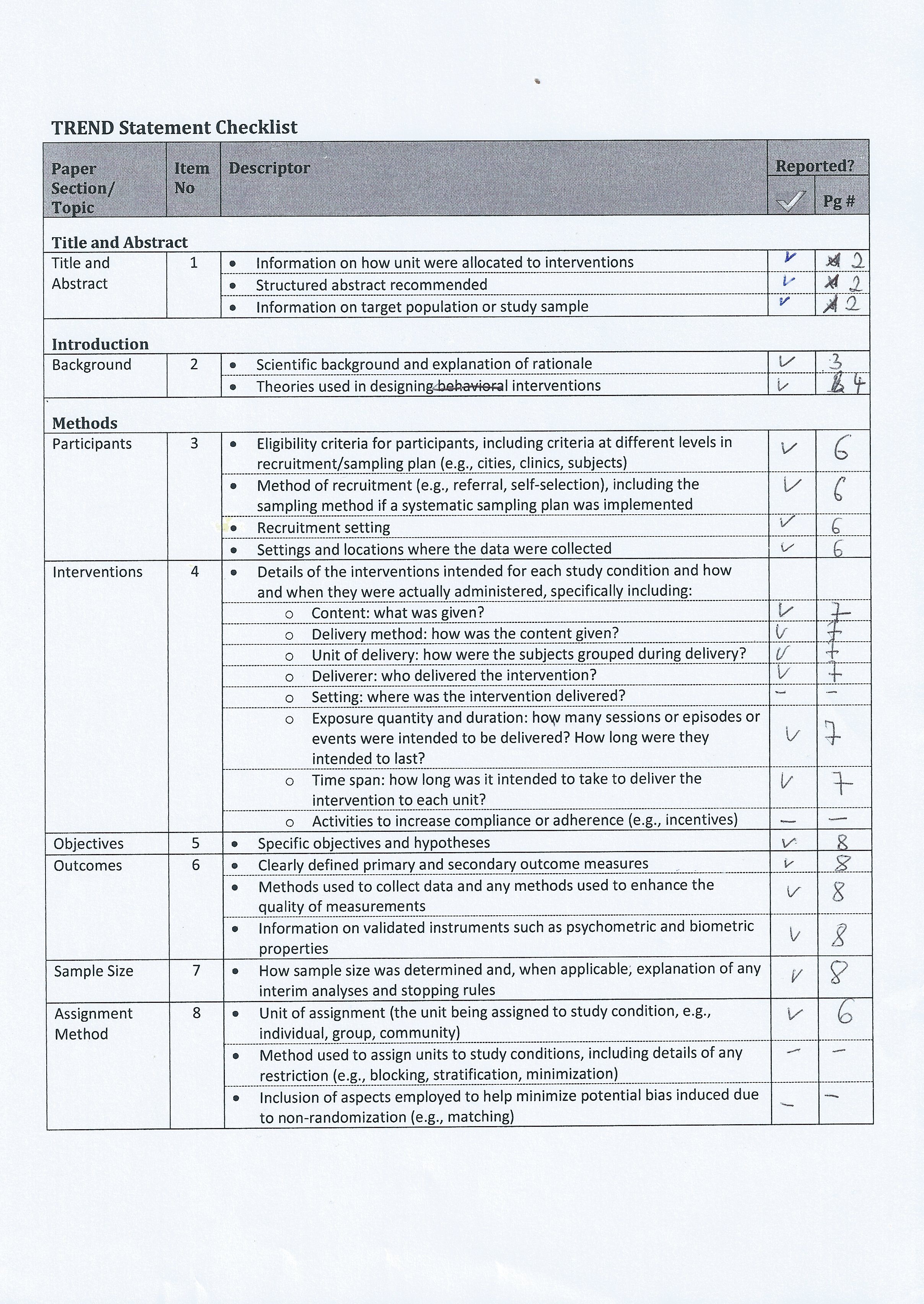

Supplement: S2 File — (JPG) [file pone.0168215.s002.jpg]

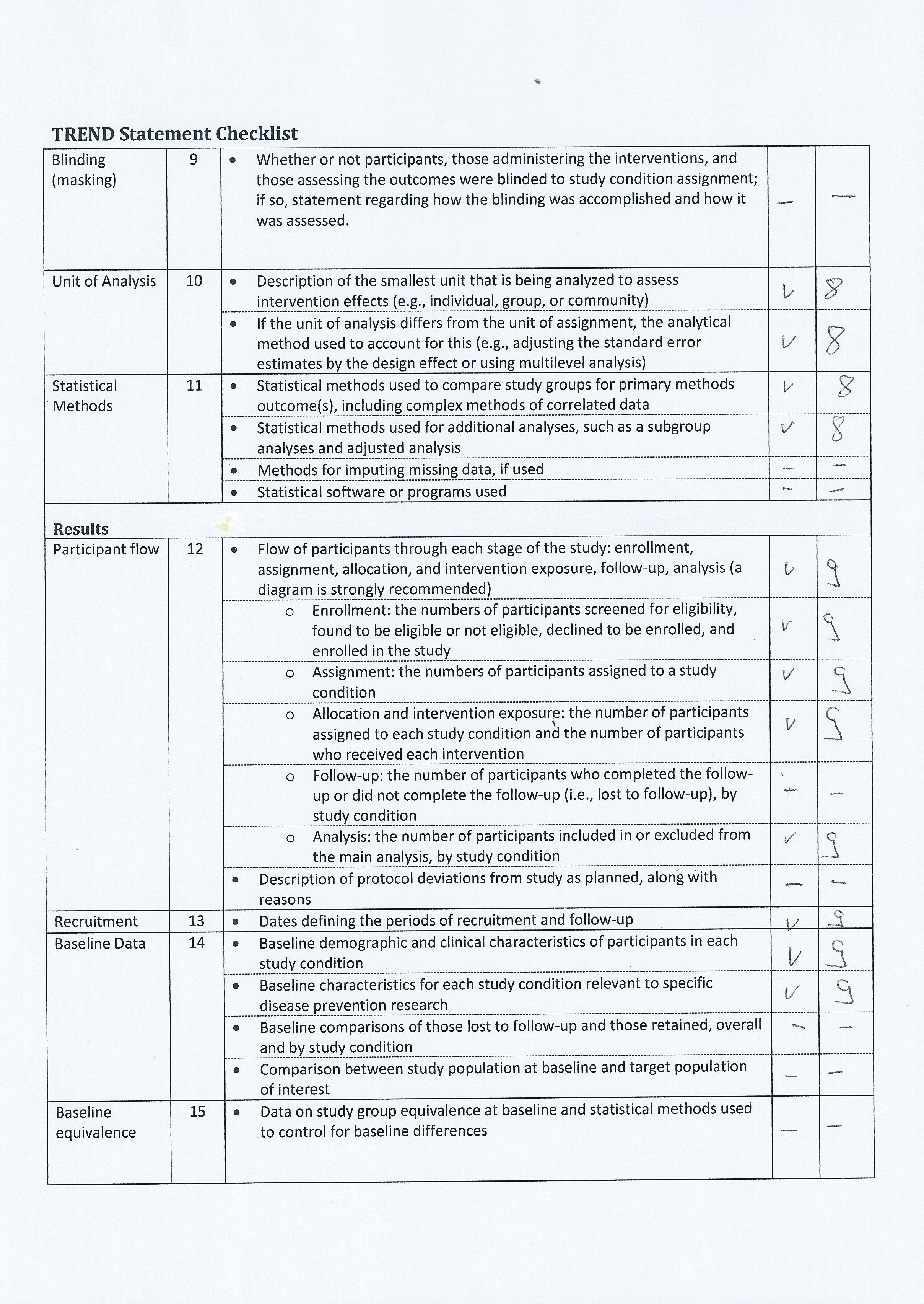

Supplement: S3 File — (JPG) [file pone.0168215.s003.jpg]

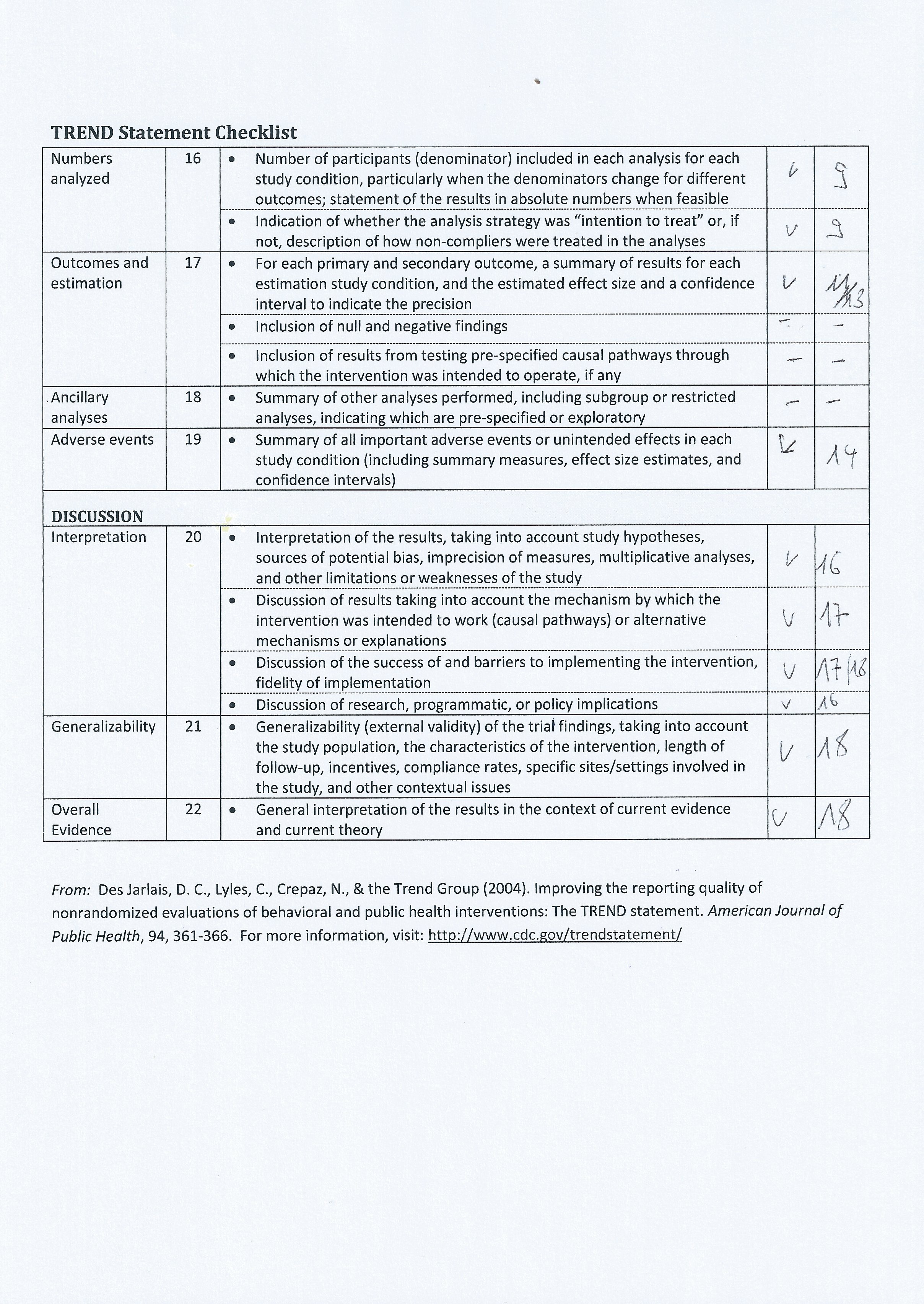

Supplement: S4 File — (JPG) [file pone.0168215.s004.jpg]
